# Hidden in the fog: morphological and molecular characterisation of *Derogenes varicus sensu stricto* (Trematoda, Derogenidae) from Sweden and Norway, and redescription of two poorly known *Derogenes* species[Fn FN1]

**DOI:** 10.1051/parasite/2023030

**Published:** 2023-09-15

**Authors:** Chahinez Bouguerche, Daniel C. Huston, Thomas H. Cribb, Egil Karlsbakk, Mohammed Ahmed, Oleksandr Holovachov

**Affiliations:** 1 Department of Zoology, Swedish Museum of Natural History Box 50007 10405 Stockholm Sweden; 2 Australian National Insect Collection, National Research Collections Australia, CSIRO PO Box 1700 Canberra ACT 2601 Australia; 3 The University of Queensland, School of Biological Sciences St Lucia QLD 4072 Australia; 4 Department of Biological Sciences, University of Bergen 7803 5020 Bergen Norway

**Keywords:** *Derogenes varicus*, *cox*1, Sweden, *Derogenes minor*, *Derogenes ruber*

## Abstract

*Derogenes varicus* (Müller, 1784) is widely reported as a trematode with exceptionally low host specificity and a wide, bipolar distribution. However, several recent studies have suggested that *D*. *varicus* represents a species complex and based on molecular evidence, four genetic lineages (labeled as “DV1–4”) have been designated within the *D. varicus* species complex. This possibility requires improved (ideally molecular) characterisation of specimens from the type-host (*Salmo salar*) and type-locality (off Denmark). During examination of trematode parasites of fish from Scandinavian and Arctic waters (Sweden and Norway), we found specimens of *D. varicus* in the stomach of *Merlangius merlangus* off the coast of Sweden, and in *Gadus morhua* off the coast of Sweden and Norway; we compared them to *D. varicus* from the type-host, the Atlantic salmon *Salmo salar* from Norway, to verify their conspecificity. Newly generated sequences (28S rDNA, ITS2 and *cox*1) of Scandinavian and Arctic specimens consistent with *D. varicus* all formed a single clade, DV1. 28S sequences of *D. varicus* from *S. salar* from Norway, i.e., close to the Danish type locality, clustered within the DV1 clade along with sequences of *D. varicus* from various hosts including *Limanda limanda*, *G. morhua* and *Myoxocephalus scorpius* from the White Sea and the Barents Sea (Russia), without any host-related structuring. We thus consider that the lineage DV1 represents *D. varicus sensu stricto*. Additionally, specimens from *M. merlangus* had a similar morphology and anatomy to those of *D. varicus* from *L. limanda*, *G. morhua* and *M. scorpius* from T. Odhner’s collection, supporting the presence of a single species in the DV1 lineage designated herein as *D. varicus sensu stricto*. We redescribe *D. varicus sensu stricto*, add new morphological characters and provide morphometric data. We infer that *D. varicus* types DV2–4 all relate to separate species. We also revise type-specimens of *Derogenes minor* Looss, 1901 from the A. Looss collection in the Swedish Museum of Natural History and provide redescriptions of it and of the type-species of the genus, *Derogenes ruber* Lühe, 1900. In light of their morphological distinctiveness relative to *D. varicus sensu stricto,* we reinstate *D. parvus* Szidat, 1950 and *D. fuhrmanni* Mola, 1912.

## Abstract (fr)

*Derogenes varicus* (Müller, 1784) est largement signalé comme un trématode avec une spécificité d'hôte exceptionnellement faible et une large distribution bipolaire. Cependant, plusieurs études récentes ont suggéré que *D. varicus* représente un complexe d'espèces. Cette possibilité nécessite une meilleure caractérisation (idéalement moléculaire) des spécimens de l'hôte-type (*Salmo salar*) et de la localité-type (au large du Danemark). Lors de l'examen des trématodes parasites des poissons des eaux scandinaves et arctiques (Suède et Norvège), nous avons trouvé des spécimens de *D. varicus* dans l'estomac de *Merlangius merlangus* au large de la Suède, et dans *Gadus morhua* au large de la Suède et de la Norvège ; nous les avons comparés à *D. varicus* de l'hôte-type, le saumon atlantique *Salmo salar* de Norvège, pour vérifier leur conspécificité. Les séquences nouvellement générées (ADNr 28S, ITS2 et *cox*1) de spécimens scandinaves compatibles avec *D. varicus* ont toutes formé un seul clade, DV1. Les séquences 28S de *D. varicus* de *S. salar* de Norvège, c'est-à-dire proches de la localité-type au Danemark, se regroupent dans le clade DV1 avec des séquences de *D. varicus* de divers hôtes dont *Limanda limanda*, *G. morhua* et *Myoxocephalus scorpius* de la mer Blanche et la mer de Barents (Russie), sans aucune structuration liée à l'hôte. Nous considérons donc que la lignée DV1 représente *D. varicus sensu stricto*. De plus, les spécimens de *M. merlangus* avaient une morphologie et une anatomie similaires à celles de *D. varicus* de *L. limanda*, *G. morhua* et *M. scorpius* de la collection de T. Odhner, confirmant la présence d'une seule espèce dans la lignée DV1 désignée ici comme *D. varicus sensu stricto*. Nous redécrivons *D. varicus sensu stricto*, ajoutons de nouveaux caractères morphologiques et fournissons des données morphométriques. Nous en déduisons que les types DV2-4 de *D. varicus* se rapportent tous à des espèces distinctes. Nous révisons également les spécimens-types de *Derogenes minor* Looss, 1901 de la collection d’A. Looss du Muséum Suédois d'Histoire Naturelle, et fournissons des redescriptions de cette espèce et de l'espèce-type du genre, *Derogenes ruber* Lühe, 1900. À la lumière de leur distinction morphologique par rapport à D*. varicus sensu stricto*, nous rétablissons *D. parvus* Szidat, 1950 et *Derogenes fuhrmanni* Mola, 1912.

## Introduction

**Figure 1:**
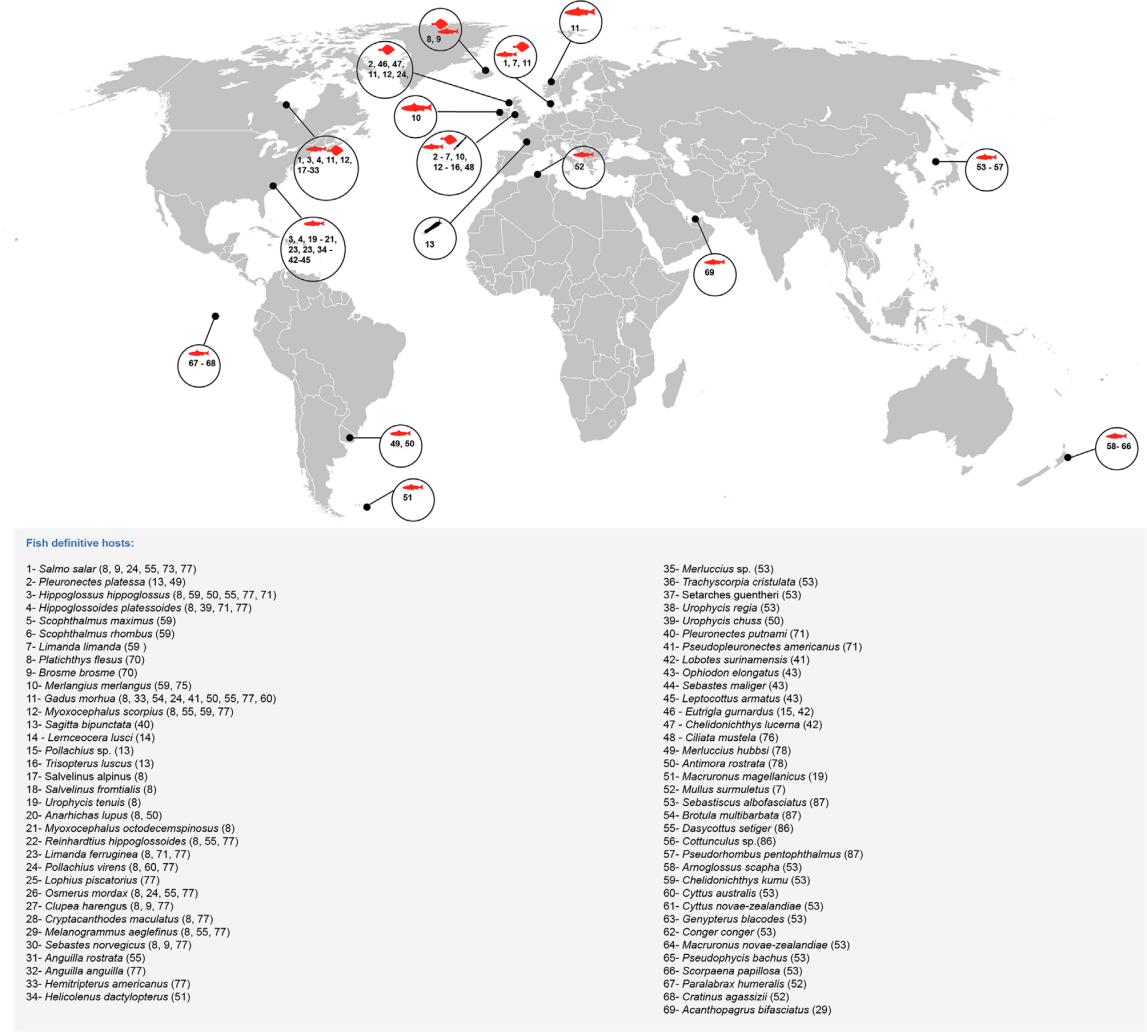
Geographical distribution of *Derogenes varicus* (Müller, 1784) from fish around the world. References for the records are given between parentheses. Note that this is not an exhaustive list of all reported hosts, excluding those for which evidence is partly lacking and illustrates mainly the numerous reports and the taxonomic range of hosts.

Some reports on digeneans from Scandinavian fishes began as early as 1784, with the description of *Fasciola varica* Müller, 1784, now known as *Derogenes varicus* (Müller, 1784), from Atlantic salmon *Salmo salar* supposedly from Danish waters [58]. This trematode warrants more attention as it is considered the most common digenean species in fish [32]. It is widely distributed in most of the oceans of the world, with a continuous three-dimensional distribution from the Arctic to the Antarctic by way of deeper waters [1, 8, 9, 13–15, 19, 24, 29, 33, 39–43, 50–55, 59, 60, 70, 71, 73, 75–78, 86, 87]. It occurs in what may be the widest range of bony fishes for any trematode species (Fig. 1).

Several authors have questioned the reported broad distribution and the lack of host specificity of *D. varicus* and have suggested that *D. varicus* comprises more than one species [20, 34]. As a result, Køie [34] proposed to designate the northeast Atlantic specimens “*D. varicus* *sensu lato*” or “*Derogenes* sp.”. That *D. varicus* is a species complex was confirmed recently by Krupenko *et al.* [35] who, based on molecular evidence, suggested the presence of four genetic lineages (designated as “DV1–4”) within the species. The vertebrate hosts of the adult stages were as follows: *Derogenes* cf. *varicus* DV1 from the White Sea, Barents Sea (Russia) and North Sea (Sweden and Norway), a euryxenous species, occurring in the Atlantic wolffish *Anarhichas lupus*, the Pacific herring *Clupea pallasii*, Navaga *Eleginus nawaga*, the cod *Gadus morhua*, the common dab *Limanda limanda*, the shorthorn sculpin *Myoxocephalus scorpius* and the Moustache sculpin *Triglops murrayii*; *Derogenes* cf. *varicus* DV2, known only from the American plaice *Hippoglossoides platessoides* from the North Sea and from gastropod hosts *Amauropsis islandica*, *Euspira pallida* and *Buccinum scalariforme* from the White Sea and Barents Sea (Russia); *Derogenes* cf*. varicus* DV3 known only from the Sea of Okhotsk, Pacific, from Fedorov’s lumpsucker *Eumicrotremus fedorovi*; and *Derogenes* cf. *varicus* DV4 known only from *Hippoglossoides platessoides* from the North Sea [35, 63].

In the course of parasitological examination of trematodes of marine fish from Scandinavian waters (Sweden and Norway) and the Arctic Ocean (Norway), we found specimens resembling *D. varicus* in the stomach of *Merlangius merlangus* off the coast of Sweden, and in *Gadus morhua* from Sweden and Norway, hosts from which *D. varicus* was previously recorded in the North Sea. We compared these with *D. varicus* from the Atlantic salmon *Salmo salar*, to confirm their conspecificity.

Here, we provide a morphological redescription of *D. varicus* *sensu stricto* and confirm its lack of host specificity using molecular sequences (28S ribosomal RNA, Internal transcribed spacer ITS2, and Cytochrome c oxidase I *cox*1 sequences). Additionally, we studied the type-material of *D. minor* Looss, 1901 from the brown wrasse, *Labrus merula*. We designate a lectotype and paralectotypes and provide a redescription of this species. We also found in A. Looss’s collections specimens of the type-species of the genus, *D. ruber* Lühe, 1900 for which we provide an illustrated redescription. The validity of species previously synonymised with *D. varicus* is considered.

## Material and methods

### Host and parasite collection

Fish were collected from Sweden, Northeast Atlantic (Skagerrak, Kattegat and Gullmarsfjorden) and from western and Arctic Norway (Table 1).

**Table 1:** Host fishes examined from Scandinavian waters of the North Sea and the Arctic Ocean during this study.

	Sweden	Norway
Species	Skagerrak, Lysekil	Gullmarsfjorden, Kristineberg	Kattegat	Svalbard	Bremanger
*Salmo salar*					+
*Merlangius merlangus*	+	+	+		
*Gadus morhua*		+	+	+	+

**Table 2:** Collection data for 28S sequences analysed in this study. A.O., Arctic Ocean. B.S., Barents Sea. M., Mediterranean. N.S., North Sea. W.S., White Sea.

Species	Host	Location	GenBank ID			Source	Collection numbers
			28S rDNA	ITS2 rDNA	*cox*1		
*D. varicus*	*Salmo salar*	Norway, NS	OQ916455	–	–	Present study	–
*D. varicus*	*Merlangius merlangus*	Sweden, NS	OQ916442	OQ916456	OR507183	Present study	SMNH 208358
*D. varicus*	*Merlangius merlangus*	Sweden, NS	OQ916444	OQ916443	OR140909	Present study	SMNH 208356
*D. varicus*	*Merlangius merlangus*	Sweden, NS	OQ916437	OQ916452	OR140894	Present study	SMNH 208354
*D. varicus*	*Merlangius merlangus*	Sweden, NS	OQ916450	OQ916438	OR140897	Present study	SMNH 208355
*D. varicus*	*Merlangius merlangus*	Sweden, NS	OQ916440	OR507184	OR507183	Present study	SMNH 208359
*D. varicus*	*Merlangius merlangus*	Sweden, NS	OQ916445	OQ916457	OR507185	Present study	SMNH 208357
*D. varicus*	*Gadus morhua*	Sweden, NS	OQ916447	OQ916446	OR140896	Present study	SMNH 218680
*D. varicus*	*Gadus morhua*	Norway, AO	OQ916454	OQ916451	OR140779	Present study	SMNH 218681
*D. varicus*	*Gadus morhua*	Norway, AO	OQ916441	OQ916449	OR140895	Present study	SMNH 218682
*D. varicus*	*Gadus morhua*	Norway, AO	OQ916448	OQ916439	OR140832	Present study	SMNH 218679
*D. varicus* DV1	*Limanda limanda*	Russia, WS	OM761962	OM762002	OM807173	[35]	
*D. varicus* DV1	*Gadus morhua*	Russia, WS	OM761963	OM762003	OM807174	[35]	
*D. varicus* DV1	*Myoxocephalus scorpius*	Russia, WS	OM761964	OM762004	OM807175	[35]	
*D. varicus* DV1	*Anarhichas lupus*	Russia, WS	OM761965	OM762005	OM807176	[35]	
*D. varicus* DV1	*Limanda limanda*	Russia, WS	OM761966	OM762006	OM807177	[35]	
*D.* *varicus* DV1	*Eleginus nawaga*	Russia, WS	OM761967	OM762007	OM807178	[35]	
*D.* *varicus* DV1	*Limanda limanda*	Russia, WS	OM761968	OM762008	OM807179	[35]	
*D.* *varicus* DV1	*Clupea pallasii*	Russia, WS	OM761969	OM762009	OM807180	[35]	
*D.* *varicus* DV1	*Clupea pallasii*	Russia, WS	OM761970	OM762010	OM807181	[35]	
*D.* *varicus* DV1	*Triglops murrayi*	Russia, WS	OM761976	OM762016		[35]	
*D.* *varicus* DV1	*Gadus morhua*	Russia, BS	OM761971		OM807182	[35]	
*D.* *varicus* DV1	*Myoxocephalus scorpius*	Russia, BS	OM761972	OM762012	OM807183	[35]	
*D. varicus* DV1	*Myoxocephalus scorpius*	Russia, BS	OM761973	OM762013	OM807184	[35]	
*D. varicus* DV1	*Gadus morhua*	Russia, WS	OM761974	OM762014		[35]	
*D.* *varicus* DV1	*Gadus morhua*	Russia, WS	OM761975	OM762015		[35]	
*D.* *varicus* DV1	*Cryptonatica affinis*	Russia, WS		OM762024		[35]	
*D. varicus* DV2	*Buccinum scalariforme*	Russia, WS	OM761977 a	OM762017 a		[35]	
*D. varicus* DV2	*Amauropsis islandica*	Russia, WS	OM761989	OM762029		[35]	
*D. varicus* DV2	*Euspira pallida*	Russia, WS		OM762030	OM807194	[35]	
*D. varicus* DV2	*Euspira pallida*	Russia, BS		OM762031	OM807195	[35]	
*D. varicus* DV3	*Eumicrotremus fedorovi*	North Pacific	MW504598			(Sokolov *et al.*, 2021)	
*D. varicus* DV3	*Eumicrotremus fedorovi*	North Pacific	MW504599			(Sokolov *et al.*, 2021)	
*D. lacustris*	*Oncorhynchus mykiss*	Argentina			LC586095	(Tsuchida *et al.*, 2021)	
*D. lacustris*	*Salvelinus fontinalis*	Argentina			LC586094	(Tsuchida *et al.*, 2021)	
*D. lacustris*	*Percichthys trucha*	Argentina			LC586093	(Tsuchida *et al.*, 2021)	
					LC586096		
*D. lacustris*	*Galaxias maculatus*	Argentina			LC586092	(Tsuchida *et al.*, 2021)	
					LC586097		
					LC586098		
*Progonus muelleri*	*Eumicrotremus fedorovi*	North Pacific	MW507469			(Sokolov *et al.*, 2021)	
*P. muelleri*	*Eumicrotremus fedorovi*	North Pacific	MW507470			(Sokolov *et al.*, 2021)	
*P. muelleri*	*Caprella septentrionalis*	Russia, WS	MW507471			(Sokolov *et al.*, 2021)	
*P. muelleri* PM2	*Myoxocephalus scorpius*	Russia, WS	OM761978	OM762018	OM807185	[35]	
*P. muelleri* PM2	*Myoxocephalus scorpius*	Russia, BS				[35]	
*P. muelleri* PM1	*Myoxocephalus scorpius*	Russia, WS	OM761979	OM762019	OM807186	[35]	
*P. muelleri* PM1	*Myoxocephalus scorpius*	Russia, WS	OM761980	OM762020		[35]	
*P. muelleri* PM1	*Myoxocephalus scorpius*	Russia, WS	OM761981	OM762021		[35]	
*P. muelleri* PM2	*Limanda limanda*	Russia, WS	OM761982	OM762022		[35]	
*P. muelleri* PM2	*Triglops murrayi*	Russia, WS	OM761983	OM762023		[35]	
*D. lacustris*	*Galaxias maculatus*	Argentina	LC586089			(Tsuchida *et al.*, 2021)	
*D. lacustris*	*Galaxias maculatus*	Argentina	LC586090			(Tsuchida *et al.*, 2021)	
*Allogenarchopsis problematica*	*Semisulcosipra reiniana*	Japan	MH628313			(Sokolov *et al.*, 2019)	
*Genarchopsis chubuensis*	*Rhinogobius flumineus*	Japan	MH628311			(Sokolov *et al.*, 2019)	
*Genarchella* sp. 1	*Herichthys labridens*	Mexico	MK648276			(De León & Hernández-Mena, 2019)	
*Genarchella* sp. 1	*Astyanax aeneus*	Mexico	MK648277			(De León & Hernández-Mena, 2019)	
*Thometrema lotzi*	*Lepomis microlophus*	USA	KC985236			(Calhoun *et al.*, 2013)	
*Thometrema patagonica*	*Percichthys trucha*	Argentina	LC586091			(Tsuchida *et al.*, 2021)	
*Prosogonotrema bilabiatum*	*Caesio cuning*	Australia	AY222191			(Olson *et al.*, 2003)	
*Accacladocoelium macrocotyle*	*Mola mola*	Spain, M		KF687303		(Ahuir-Baraja *et al.*, 2015)	
*Didymocystis wedli*	*Thunnus orientalis*	Japan			AB725624	(Abe *et al.*, 2014)	

a Two sequences are wrongly annotated on GenBank: OM761977.1 and OM762017.1, these two *Derogenes varicus* complex sp. DV1 isolates are in fact DV2.

**Table 3:** Measurements of *Derogenes varicus sensu stricto*. NEA, Northwest Atlantic.

Species		*Derogenes varicus sensu stricto*				

Host	*Salmo salar*	*Merlangius merlangus*	*Limanda limanda*	*Myoxocephalus scorpius*	*Gadus morhua*	Several hosts^a^
Locality	Bremanger, Norway, NEA	Skagerrak, Sweden, NEA	Kristineberg, Sweden, NEA	Kristineberg, Sweden, NEA	Kristineberg, Sweden, NEA	White Sea, Barents Sea, Russia, NEA
Source	Present study	Present study	Present study	Present study	Present study	Krupenko *et al.* [35]
Number of specimens	16	23	1	2	2	–
Body L	2276 (1812–2661)	1453 (1147–1882)	2985	2192–2280	1978–2334	1291 (873–1885)
Body W	528 (392–669)	380 (216–499)	838	552–670	460 – 603	371 (257–498)
Forebody	838 (739–937) × 385 (334–418)	653 (470–858) × 342 (272–416)	1316 × 717	1087–1135 × 491–641	979–986 × 380–551	527 (349–786)
Hindbody	803 (698–930) × 382 (343×472)	516 (327–722) × 307 (153–432)	1150 × 615	766–800 × 275–477	696–936 × 224–337	104 (41–165)
Preoral lobe L	57 (44–68)	43 (23–71)	39	14	30–32	28 (20 – 40)
Ventral sucker	353 (231×399) × 374 (268×434)	300 (251–384) × 299 (251–359)	457 × 456	359–429 × 332–411	326–419 × 332–433	304 (224–426) × 313 (219–437)
Oral sucker	255 (223–335) × 247 (219–321)	164 (130–223) × 171 (132–235)	229 × 260	180–214 × 216–249	204–251 × 212–256	152 (89–214) × 167 (117–224)
Pharynx	90 (79–112) × 101 (85–135)	67 (55–81) × 81 (69–96)	114–112	79–85 × 98–115	74–76 × 81–95	65 (41–87) × 77 (52–104)
Sinus-sac	120 (93–135) × 113 (106–120)	104 (83–131) × 92 (78–112)	–	–	102–106 × 103–123	84 (64–112) × 87 (60–106)
Seminal vesicle	70 (46–80) × 45 (30–75)	72 (48–85) × 46 (35–55)	168–68	–	96–107 × 40–41	75 (39 – 225) × 46 (23–81)
Left testis	180 (152–208) × 145 (128–165)	122 (97–171) × 109 (87–148)	108–121	143–146 × 107–123	979–986 × 380–551	105 (70–157) × 105 (62–144)
Right testis	164 (124–208) × 147 (120–180)	130 (102–202) × 119 (95–174)	109–120	98–117 × 119–125	119–130 × 104–141	105 (75–142) × 102 (61–160)
Ovary	151 (128–180) × 155 (112–188)	120 (97–152) × 116 (104–143)	157 × 212	124–159 × 159–128	101–134 × 123–136	103 (77–153) × 109 (69–158)
Left vitellarium	228 (201–256) × 176 (136–207)	164 (123–189) × 148 (94–204)	232 × 192	183–211 × 163–188	135–212 × 112–144	152 (109–203) × 116 (63–201)
Right vitellarium	216 (181–280) × 162 (133–185)	174 (111–225) × 153 (131–173)	233 × 159	202–226 × 111–189	154–214 × 113–181	144 (97–211) × 114 (69–173)
Eggs	50 (40–53) × 31 (28–35)	52 (48–58) × 34 (30–52)	51 × 31	53–57 × 38–39	46–58 × 30–35	51 (44–60) × 31 (25–37)

a For complete hosts list, see Krupenko *et al.* [35]. L., length. W., width.

**Table 4:** Measurements of *Derogenes ruber* Lühe, 1900 and *Derogenes minor* Looss, 1901*.* M., Mediterranean. L., length. W., width. R., ratio.

Species	*D. ruber*	*D. ruber*	*D. ruber*	*D. minor*	*Derogenes* cf. *minor*
Host	*Trigla lyra*	*Ch. lastoviza as T. lineata*	*Ch. lastoviza as T. lineata*	*Labrus merula*		*Lophius piscatorius*	
Habitat	Gall-bladder	Gall-bladder	Intestine	Intestine	Intestine	Intestine	Intestine
Locality	Off Split, Croatia, Western M	Off Split, Croatia, Western M	Off Trieste, Italy, Central M	Trieste, Italy, Central M	Trieste, Italy, Central M	Trieste, Italy, Central M	
Number of specimens	2	2	2	12	1	1	1
Source	[74]	[47]	Present study	[45]	Paratype, Present study	Present study	Present study
Body L	4200–4500	5000–6000	7869	Max 2000	2022	–	2412
Body W	1300–1800	2000	1847	460	477	787	675
Forebody			3765 × 1714		983 × 500	813 × 720	1200 × 631
Hindbody			2750 × 959		706 × 366	– × 655	836 × 500
Pre-oral lobe			88		22	53	
Oral sucker	505 × 505	600*	655 × 708	220*	222 × 207	318 × 281	226 × 250
Ventral sucker	950–1290	750*	1257 × 1230	330*	349 × 321	432 × 398	358 × 391
Pharynx	168	200	254 × 275	90*	91 × 96		
Seminal vesicle			291 × 134		119 × 32		79 × 106
Pars prostatica							
Right testis	252 × 168		271 × 353		136 × 101		90 × 105
Left testis	196 × 140		256 × 280		115 × 88		79 × 106
Sinus - organ					97 × 92		
Ovary	252 × 252				129 × 107		148 × 177
Right vitelline mass	440 × 420	450	546 × 658		204 × 109	252 × 156	256 × 176
Left vitelline mass	470 × 440		550 × 446		180 × 103	262 × 255	298 × 190

Eggs	623 × 23	56–36	62 × 39	60 × 38	58 × 39	59 × 36	60 × 40

* Diameter.

Specimens from Skagerrak and Kattegat were collected during the biannual International Bottom Trawl Survey by the SLU Aqua team as part of the International Bottom Trawl Survey along the Swedish coast within the scope of their research projects and permits. Specimens were euthanized and made available for examination. Specimens from Gullmarsfjorden were collected in the vicinity of Kristineberg Center for Marine Research and Innovation, outside of the borders of the Gullmarns nature reserve, and within the scope of the permit for animal research from the Swedish Board of Agriculture (Enheten för försöksdjur och sällskapsdjur, Jordbruksverket, Dnr. 5.2.18-5483/18) and ethical approval for animal research from the Uppsala animal ethics committee (Uppsala djurförsöksetiska nämnd, Jordbruksverket, Dnr. 5.8.18-17209/2021) issued to the Swedish Museum of Natural History. Specimens from the Arctic Ocean were collected during the HHUMTL22 cruise by the Arctic University Museum of Norway, within the scope of the fieldwork sampling permit issued by the governor of Svalbard (RiS-1D12021Al) and the permission to trawl from the Norwegian Directorate of Fisheries (21/16250).

Digeneans were collected from freshly killed fish. The gastrointestinal tract was removed and examined for trematodes using the gutwash method [12, 28]. Trematodes were heat-killed, fixed without pressure in near-boiling saline, and preserved immediately in 80% ethanol for parallel morphological and molecular characterization. Ten specimens were processed as hologenophores (*sensu* [66]).

### Morphological methods

Whole-mounts for morphological analysis were stained with acetocarmine or paracarmine, dehydrated in a graded ethanol series, cleared in clove oil, and mounted in Canada balsam. The hologenophores were processed according to the same methods. Drawings were made through a Nikon Eclipse i80 microscope with DIC (differential interference contrast) and a drawing tube. Drawings were scanned and redrawn on a computer with Adobe Illustrator 2022.

Measurements of whole-mounts and of hologenophores are in micrometres and indicated as the range followed by the number of measurements in parentheses. The following abbreviation is used: SMNH, Swedish Museum of Natural History, Stockholm, Sweden.

### Molecular methods

Genomic DNA was extracted from a total of 10 hologenophores, and genetic sequence data were generated for three markers: a partial region of the *cox*1 mitochondrial region (*cox*1 mtDNA), the second internal transcribed spacer region (ITS2 rDNA), and the large (28S) ribosomal subunit RNA coding region. Polymerase chain reactions for all markers were performed in 25 μL of a mixture using an Illustra Hot Start Mix RTG 0.2 mL reaction kit (GE Healthcare Life Sciences, Uppsala, Sweden). The reaction mix consisted of 1 μL (0.4 μM) of each primer, 2 μL template DNA and 21 μL nuclease-free water. A fragment of the cytochrome oxidase I gene (*cox*1) was amplified using the primers JB3 (5′–TTTTTTGGGCATCCTGAGGTTTAT–3′) and COI R-Trema (5′–CAACAAATCATGATGCAAAAGG–3′) [84]. The reaction conditions were 4 min at 96 °C; 35 cycles of (10 s at 98 °C, 30 s at 45 °C, 1 min at 72 °C) and a final extension for 5 min at 72 °C. The 28S region was amplified using the primers 502 5′–CAAGTACCGTGAGGGAAAGTTGC–3′ [17] and 536 5′–CAGCTATCCTGAGGGAAAC–3′ [65] with the following reaction conditions: 3 min at 94 °C, 35 cycles (60 s at 94 °C, 60 s at 54 °C, 1 min at 72 °C) and a final extension for 7 min at 72 °C. For the ITS2 rDNA region, the primer pair 3S 5′–GGTACCGGTGGATCACGTGGCTAGTG–3′ [57] and ITS2.2 5′–CCTGGTTAGTTTCTTTTCCTCCGC–3′ [11] were used. The PCR conditions were as follows: a single cycle of 3 min at 95 °C, 2 min at 45 °C, and 90 s at 72 °C, followed by 4 cycles of (45 s at 95 °C, 45 s at 50 °C, and 90 s at 72 °C), then 30 cycles of (20  s at 95 °C, 20 s at 52 °C, 90 s at 72 °C). PCR products were enzymatically purified using Exonuclease I and Shrimp Alkaline Phosphatase (New England Biolabs, Ipswich, MA, USA) and then sent out to Macrogen Europe B.V. (Amsterdam, Netherlands) for sequencing. Each amplicon was sequenced in both directions using the amplification primers. We used CodonCode Aligner version 3.7.1 software (Codon Code Corporation, Dedham, MA, USA) to edit sequences, compared them to the GenBank database content with BLAST, and deposited them in GenBank under accession numbers OQ916437–OQ916457, OR140779, OR140832, OR140894–OR140897, OR140909, OR507183–OR507185.

### Trees and distances

Phylogenetic analyses were performed using the newly generated sequences of *D. varicus* and those of closely related species available in GenBank (Table 2), mostly *D. varicus* complex and *Progonus muelleri* (Levinsen, 1881) complex provided by Krupenko *et al.* [35]. Alignments for each gene region were constructed separately in AliView [37]. The alignment was manually refined and trimmed to the shortest sequence. Nucleotide substitution models for phylogenetic analyses using the Maximum Likelihood (ML) method were selected using MEGA11 [80]. The Kimura 2-parameter model with Gamma Distributed (K2+G) model was selected for the 28S, Kimura 2-parameter (K2) model for ITS2, and Tamura-Nei model (TN93) with Gamma Distributed with Invariant sites (HKY+ G+I) for *cox*1. All trees were constructed in MEGA11, with 500 replications. The Neighbor-Joining (NJ) method [72] was also used for comparison in MEGA11, with 2,000 bootstraps computed for *cox*1, ITS2 and 28S from the same datasets. P distances [31] were computed from the same datasets with MEGA11.

## Results

### Molecular characterisation

**Figure 2:**
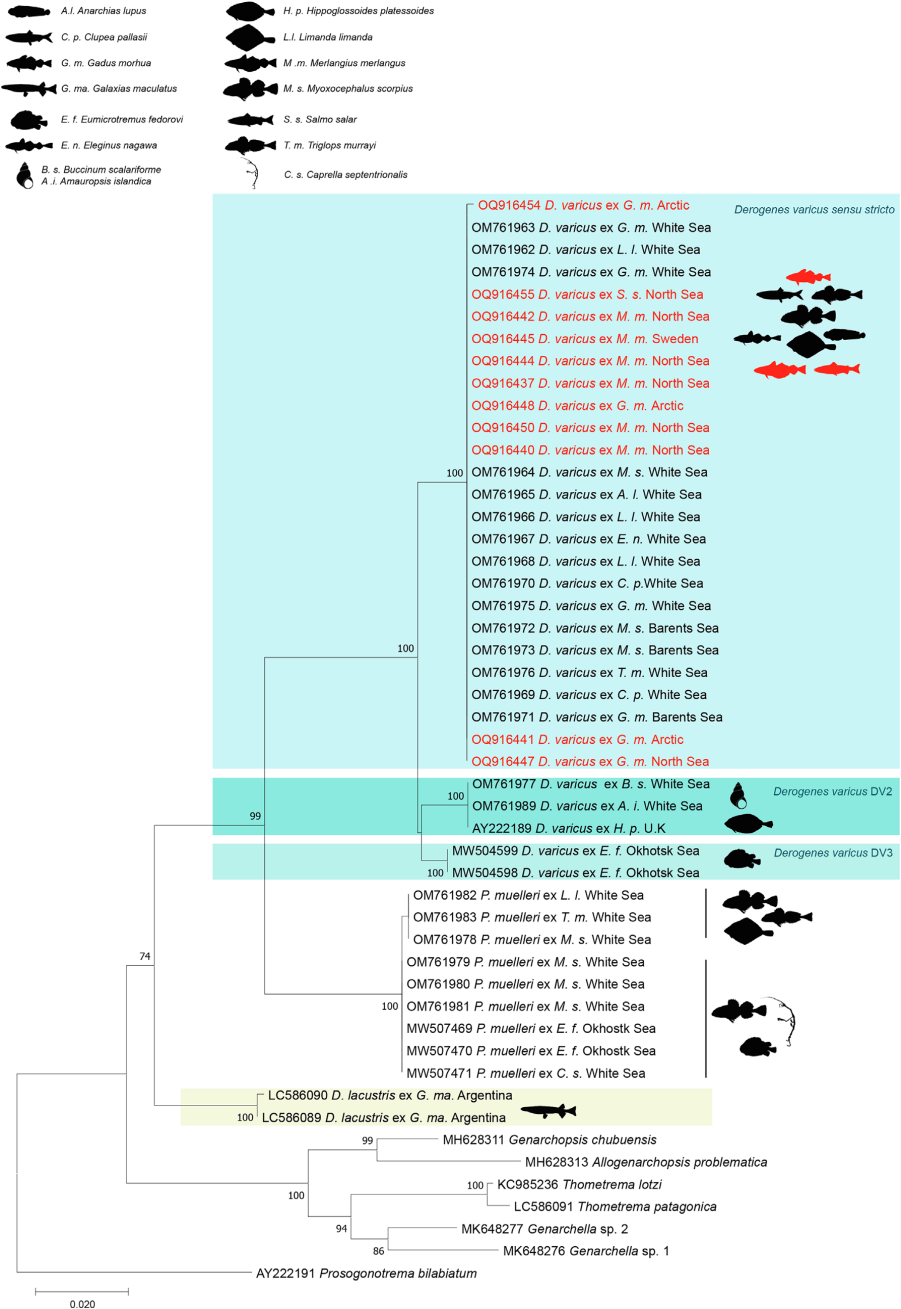
Tree inferred using the ML method based on the 28S rDNA sequence data; only bootstrap values higher than 70 are indicated. The newly generated sequences are indicated in red. Sequence of *Derogenes varicus* from the type-host is in bold. Lineages DV1, DV2, DV3 and *Derogenes lacustris* are in different colours.

**Figure 3:**
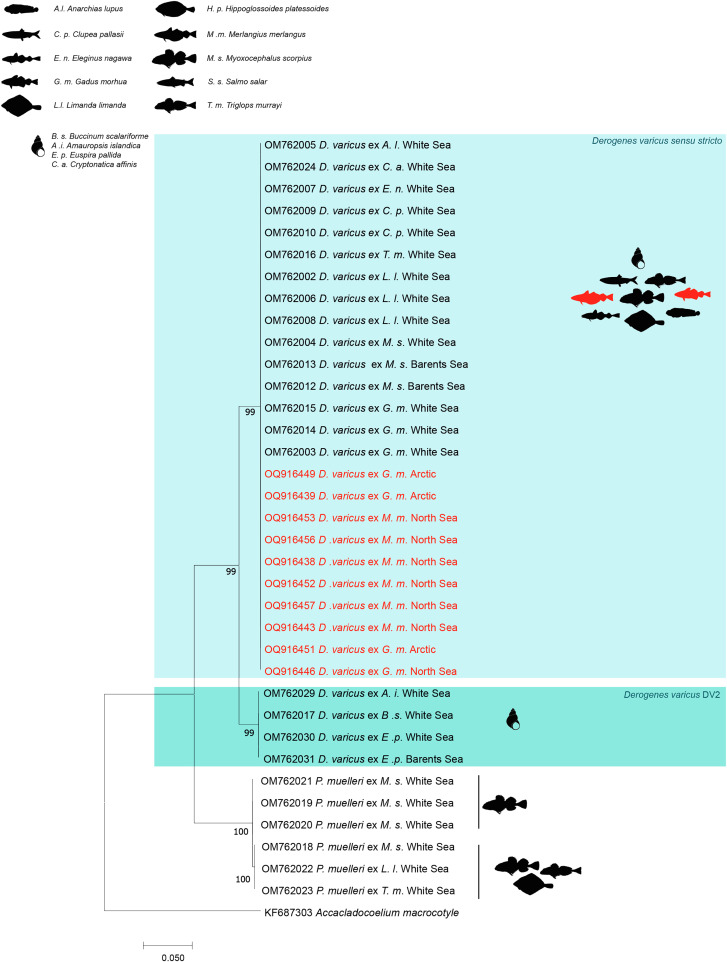
Tree inferred using the ML method based on the ITS2 rDNA sequence data; only bootstrap values higher than 70 are indicated. *Derogenes* *varicus* lineages DV1, DV2 are in different colours.

**Figure 4:**
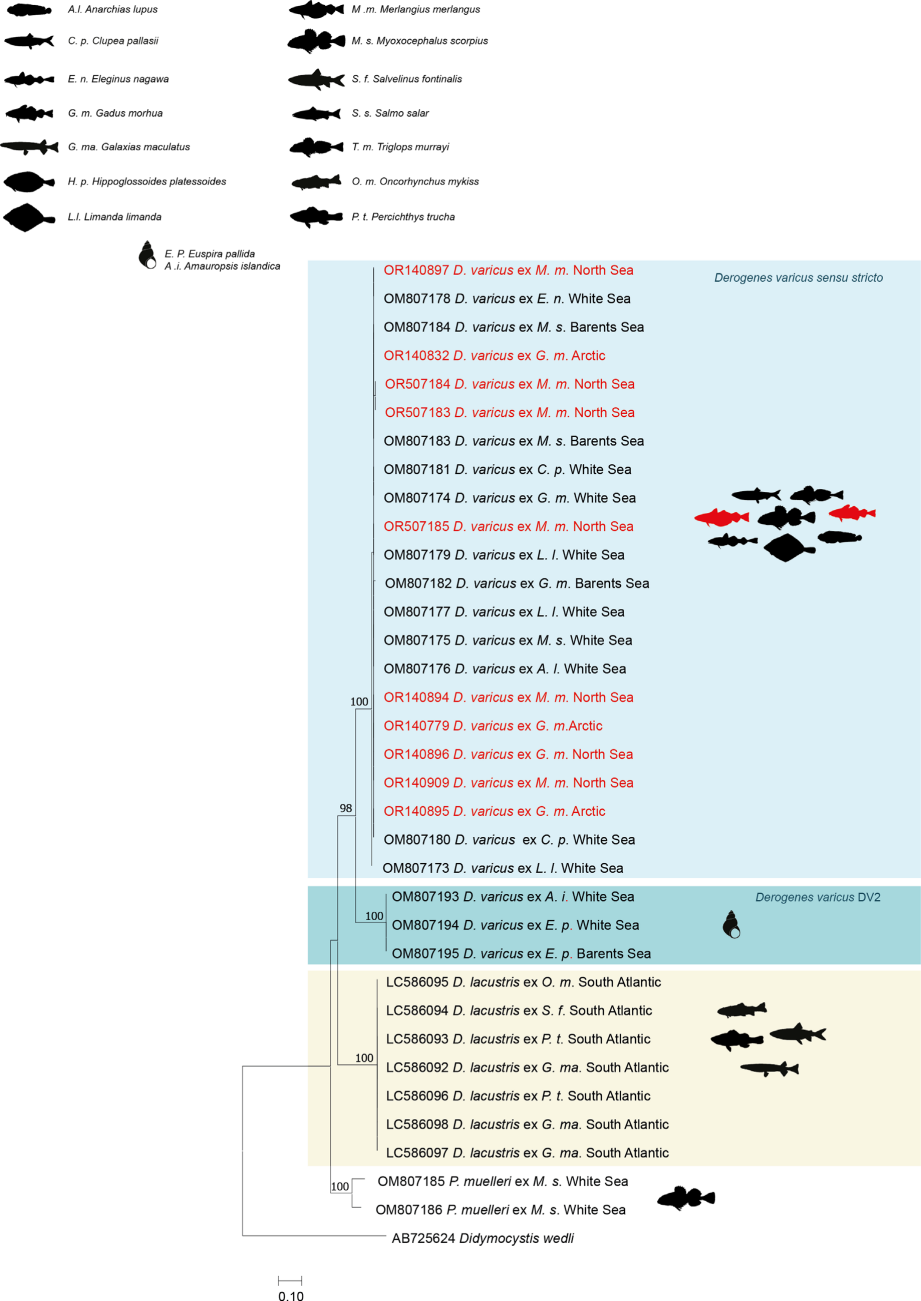
Tree inferred using the ML method based on the *cox*1 gene sequences; only bootstrap values higher than 70 are indicated. The newly generated sequences are indicated in red. Lineages DV1, DV2 *Derogenes lacustris* Tsuchida, Flores, Viozzi, Rauque & Urabe, 2021 are in different colours.

The NJ and ML methods led to similar tree topologies and thus only the ML trees are shown (Figs. 2–4).

A total of 800–857 bp of 28S rDNA were successfully sequenced for 11 individuals of *D. varicus*. The 28S dataset included 48 nucleotide sequences of derogenids. The trimmed matrix included 748 positions. The genetic divergence among the newly generated sequences was 0%. The newly generated sequences of *D. varicus* from *S. salar* from Norway and from *M. merlangus* from Sweden and those from *G. morhua* from Sweden and Norway were identical to sequences of *D. varicus* DV1 from *G. morhua*, *M. scorpius*, *L. limanda*, *A. lupus*, *E. nawaga* and *C. pallasii* from the White and Barents seas [35].

All newly generated sequences differed from those of *D.* cf. *varicus* DV2 from *H. platessoides* from the North Sea, *Amauropsis islandica* and *Buccinum scalariforme* off the coast of Russia [35] and from *D. varicus* from *Eumicrotremus fedorovi* from the North Pacific [22] by 2% (16 substitutions). The highest interspecific variation was between *D. varicus* DV1 and *D. lacustris* from *Galaxias maculatus* off the coast of Argentina [82], reaching 9% (68 substitutions). The divergence between *D. varicus* DV1 and *P. muelleri* 1 from *M. scorpius*, from *L. limanda* and from *Triglops murrayi*, and *P. muelleri* 2 from *M. scorpius* and from *E. fedorovi* collected off the coast of Russia [35] was 7%.

The newly generated 28S sequences of *D. varicus* from *G. morhua*, *M. merlangus* and *S. salar* collected off the coast of Sweden and Norway clustered as a well-supported clade (Fig. 2), with sequences designated as *D. varicus* DV1 (see [35]). This clade was well separated from the *D.* cf. *varicus* DV2 clade (from *H. platessoides*, *Amauropsis islandica* and *B. scalariforme*), the *D.* cf. *varicus* DV3 clade (from *E. fedorovi*) and the *D. lacustris* clade (from *G. maculatus*). Sequences of *D. varicus* *sensu stricto* from the type-host *S. salar* from Norway clustered within the *D. varicus* DV1 clade, without any host-related structuring, and we refer to the DV1 clade of Krupenko *et al.* [35] as “*D. varicus sensu stricto*”. Another clade with high support included *P. muelleri* 1 (from *M. scorpius*, from *L. limanda* and from *T. murrayi*) and *P. muelleri* 2 (from *M. scorpius* and from *E. fedorovi*). The sub-clades relating to *P. muelleri* 1 and *P. muelleri* 2 were not well supported.

A total of 434–562 bp of ITS2 were successfully sequenced for the same 10 individuals of *D. varicus* as for 28S. The ITS2 tree was constructed using 35 sequences of derogenids (Fig. 3). The trimmed matrix included 429 positions. The newly generated ITS2 sequences of *D. varicus* from *M. merlangus* and from *G. morhua* from Scandinavian waters and those of *D. varicus* DV1 from the previously mentioned hosts were identical. Sequences of *D. varicus* DV1 differed from *D.* cf. *varicus* DV2 from the gastropods *Amauropsis islandica*, *B. scalariforme* and *Euspira pallida* off the coast of Russia [35] by 4%.

The newly generated ITS2 sequences of *D. varicus* from *M. merlangus* and from *G. morhua* from Scandinavian waters and *D. varicus* DV1 from the previously mentioned hosts clustered within a well-supported clade, separated from the *D.* cf. *varicus* DV2 clade. Within the *P. muelleri* clade collected off the coast of Russia [35], *P. muelleri* 1 subclade (from *M. scorpius*, from *L. limanda* and from *T. murrayi*) was well supported relative to the *P. muelleri* 2 subclade (from *M. scorpius*).

The *cox*1 sequences of *D. varicus* were aligned with 24 other derogenid sequences, all relating to the genera *Derogenes* and *Progonus*. The trimmed matrix included 781 positions. The divergence between the newly generated sequences was 0–1% (1 substitution). Sequences of *D. varicus* from *M. merlangus* and from *G. morhua* from Scandinavian waters differ from those of *D. varicus* DV1 from *G. morhua*, *M. scorpius*, *L. limanda*, *A. lupus*, *E. nawaga* and *C. pallasii* off the coast of Russia [35] also by 0–1% (1 substitution). The divergence between *D. varicus* DV1 and sequences of *D.* cf. *varicus* DV2 (from *E. pallida* off the coast of Russia [35]) and from *D. lacustris* (from *G. maculatus*, *P. trucha*, *S. fontinalis* and from *O. mykiss*, off the coast of Argentina [82]) were 15–16% (131 substitutions) and 19% (145 substitutions), respectively. Divergence between *D. varicus* DV1 and *P. muelleri* from *M. scorpius* from off the coast of Russia [35] ranged between 18% and 21%.

All newly generated *cox*1 sequences of *D. varicus* clustered with those of *D. varicus* DV1 within a well-supported clade (Fig. 4), well separated from that of *D.* cf. *varicus* DV2 and from the *D. lacustris* clade. Another clade with strong support included two sequences of *P. muelleri* from *M. scorpius* which nested as sister clade to the *Derogenes* clade.

### Morphology

Family Derogenidae Nicoll 1910

Subfamily Derogeninae Nicoll 1910

Genus *Derogenes* Lühe 1900

#### *Derogenes varicus* (Müller, 1784) sensu stricto (Figs. 5–8)

**Figure 5:**
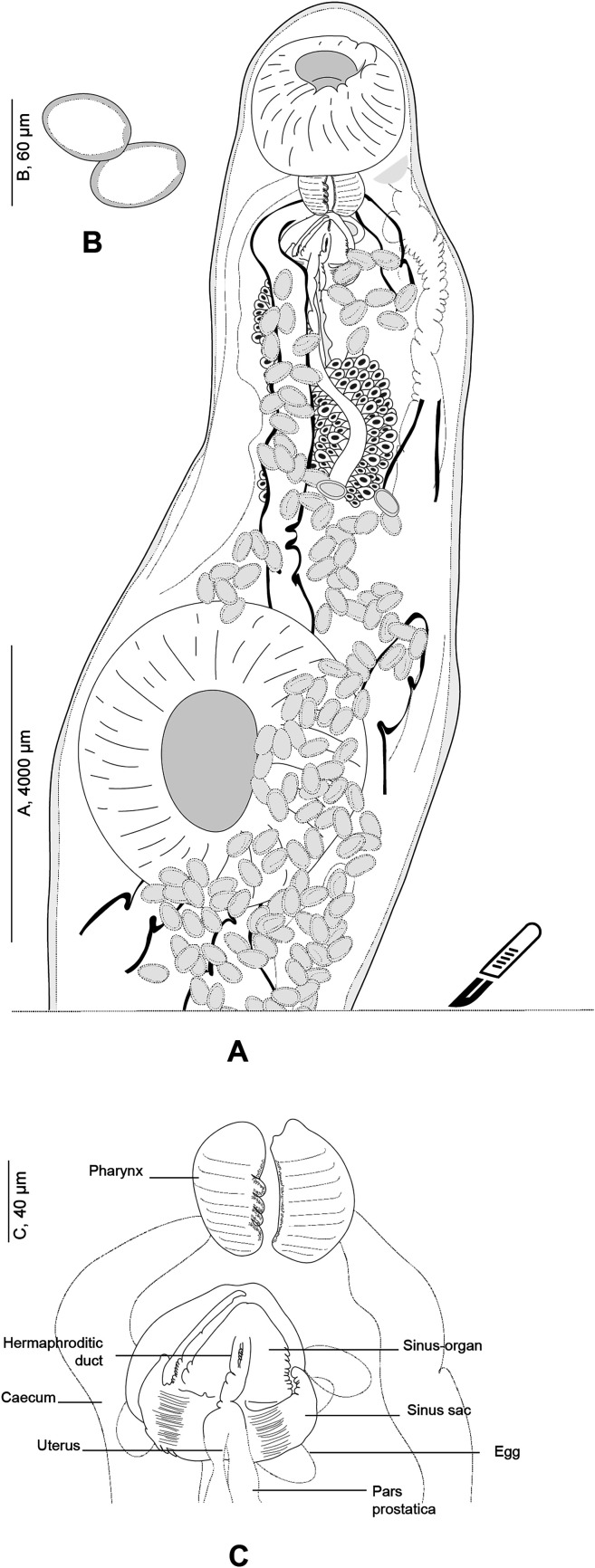
*Derogenes varicus* (Müller, 1784) *sensu stricto* ex *Gadus morhua* (SMNH 218681), Hologenophore. A, Body. B, Egg. C, Terminal genitalia.

**Figure 6:**
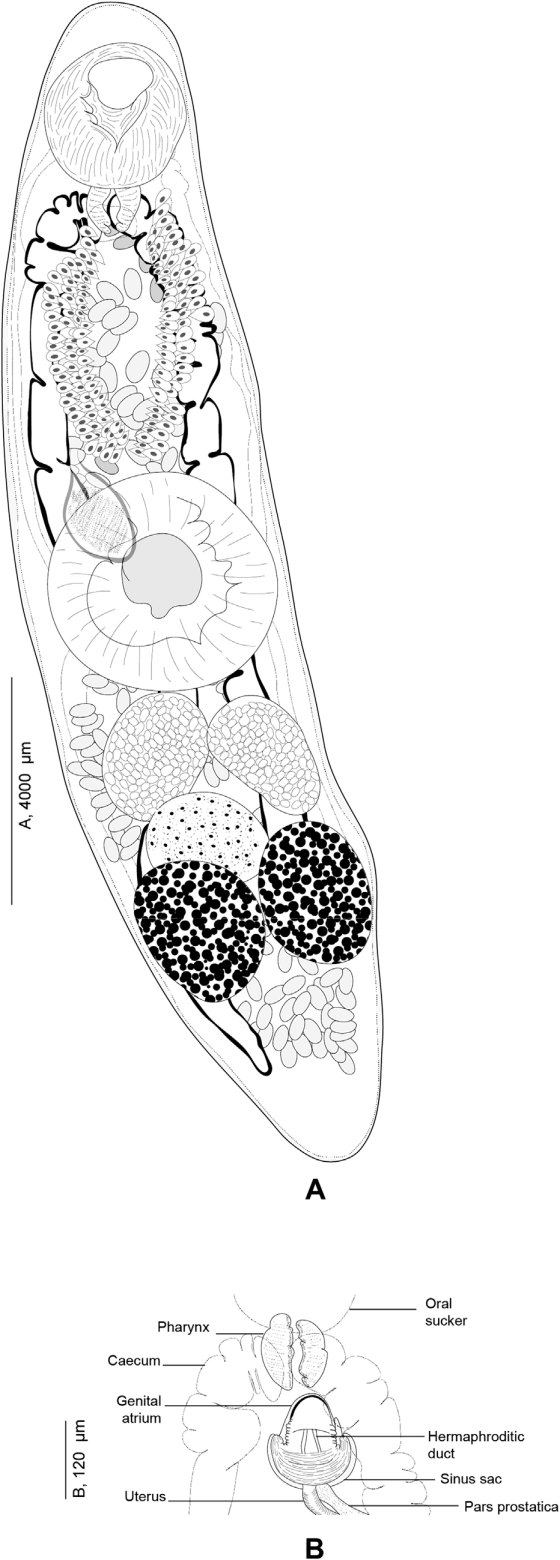
*Derogenes varicus* (Müller, 1784) *sensu stricto* ex *Salmo salar* (SMNH 218683, SMNH 218684)*.* A, Whole body (SMNH 218683). B, Terminal genitalia (SMNH 218683). C, Posterior part showing excretory vesicle (SMNH 218684).

**Figure 7:**
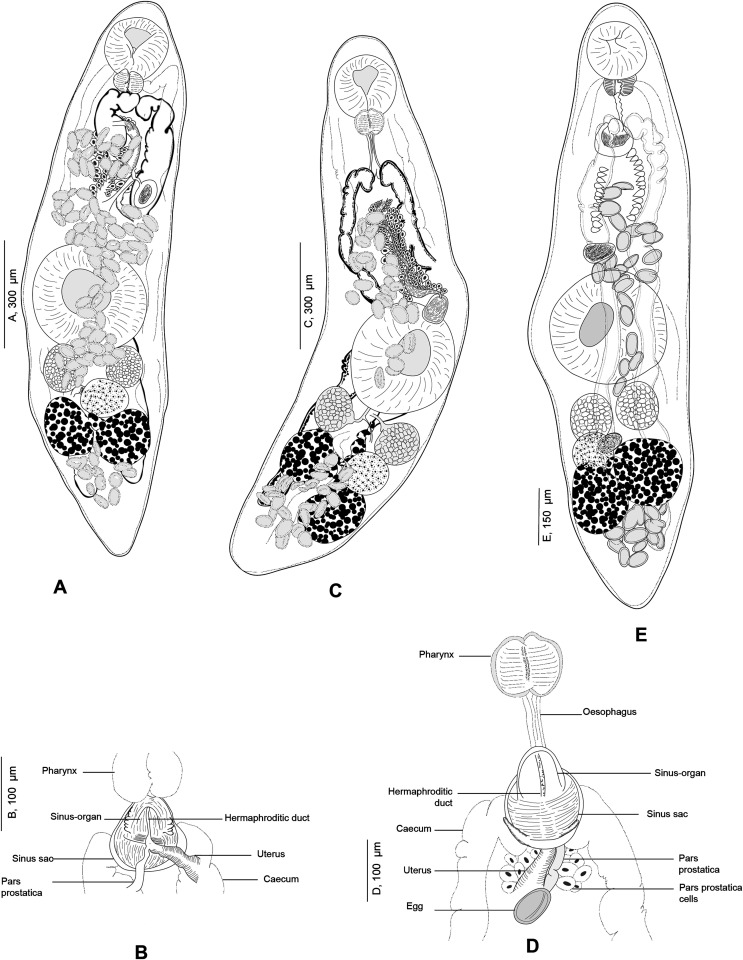
*Derogenes varicus* (Müller, 1784) *sensu stricto* ex *Merlangius merlangus*. A, Whole body (SMNH 218721). B, Terminal genitalia (SMNH 218721). C, Whole body of a flattened specimen (SMNH 218722). D, Terminal genitalia of a flattened specimen (SMNH 218722). E, Whole body (SMNH 218723).

**Figure 8:**
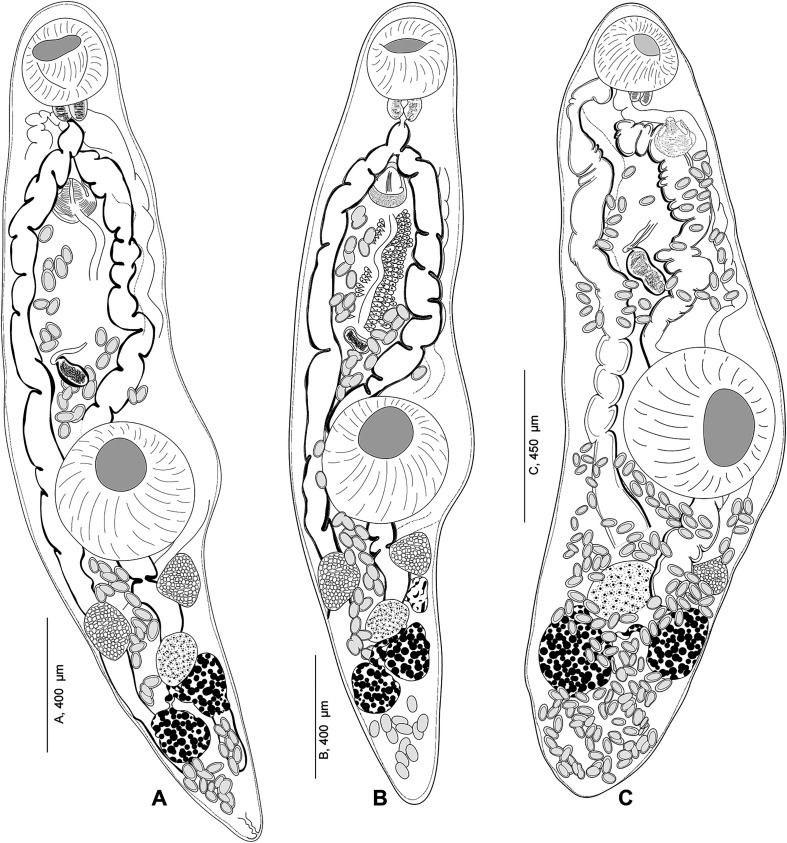
*Derogenes varicus* (Müller, 1784) *sensu stricto* from different hosts. A, Whole body ex *Myoxocephalus scorpius* (SMNH-114553). B, Whole body ex *Gadus morhua* (SMNH-1145522). C, Whole body ex *Limanda limanda* (SMNH-1145511).

Type-host: *Salmo salar* (Salmoniformes: Salmonidae), the Atlantic salmon [58].

Type-locality: off the coast of Denmark, Northeast Atlantic [58].

Site in host: Stomach.

Additional hosts (only those confirmed by DNA barcodes): *Anarhichas lupus* (Anarhichadidae), Atlantic wolffish; *Clupea pallasii* (Clupeidae), Pacific herring [35]; *Myoxocephalus scorpius* (Cottidae), shorthorn sculpin [35] (present study); *Triglops murrayi* (Cottidae), moustache sculpin; *Eleginus nawaga* (Gadidae), navaga [35]; *Gadus morhua* (Gadidae), Atlantic cod; *Limanda limanda* (Pleuronectidae), common dab [35] (present study). For invertebrate hosts see Krupenko *et al.* [35].

Additional localities: Kristineberg, Sweden, Northeast Atlantic (present paper, specimens found in T. Odhner’s collections in the SMNH). Skagerrak, Kattegat, Sweden, Northeast Atlantic, present paper. Bremanger, Norway, Northeast Atlantic, present paper. Svalbard, Norway, Arctic Ocean, present paper. Keret Archipelago; Velikaya Salma Strait, White Sea and Dalniye Zelentsy, Barents Sea [35].

Specimens deposited: Specimens with molecular information: anterior parts of specimens mounted on slide, posterior part used for molecular analysis: specimens from *Merlangius merlangus* off the coast of Sweden; SMNH 208354-208359. Specimens from *Gadus morhua* off the coast of Sweden; SMNH 218680. Specimens from *Gadus morhua* off the coast of Norway; SMNH 218679, SMNH 218681, SMNH 218682.

Specimens examined for morphological study, whole mounts: *D. varicus* from *Salmo salar* from Bremanger, Norway, North Atlantic (SMNH 218683-218700); *D. varicus* from *Gadus morhua* from Norway, Arctic Ocean (SMNH 218701-218708) and from Sweden, North Atlantic (SMNH 218709-218720); *D. varicus* from *merlangius merlangus* from Norway, from Sweden, North Atlantic (SMNH 218721-218734).

Material examined for comparison: *Derogenes* *varicus*: 1 specimen of *D. varicus* from *L. limanda* (SMNH-114551), 2 specimens of *D. varicus* from *G. morhua* (SMNH-114552), 2 specimens of *D. varicus* from *M. scorpius* (SMNH-114553), from Kristineberg, Sweden, Northeast Atlantic; from the collection of T. Odhner deposited in the Invertebrates collection in the SMNH.

##### Description

Based on 23 specimens. Measurements in Table 3. Body elongate, sausage-shaped, with maximum width occurring at level of ventral sucker. Anterior extremity rounded; posterior extremity slightly pointed. Tegument smooth, uniformly thick.

Pre-oral lobe short, surmounting oral sucker. Oral sucker rounded. Prepharynx absent. Pharynx small, muscular, sub-globular to stocky. Oesophagus short. Intestinal bifurcation in anterior third of forebody. Intestinal caeca terminating blindly posteriorly to vitelline masses. Ventral sucker round, voluminous, generally as long as wide, located at level of mid-body.

Testes globular, symmetrical to slightly oblique, immediately posterior to ventral sucker. Seminal vesicle oval, muscular and thin-walled, located usually anterior to ventral sucker with position affected by state of contraction of specimens. Pars prostatica relatively long, difficult to trace in unflattened specimens, lined by numerous gland cells, leading to seminal vesicle. Sinus-sac small, oval. Ejaculatory duct and metraterm visible passing into sinus-sac then into sinus-organ forming hermaphroditic duct. Hermaphroditic duct tubular thin-walled. Sinus-sac present. Cone-shaped permanent muscular sinus-organ projecting into genital atrium. Genital pore transversely oval, ventral, posterior to pharynx.

Ovary oval, posterior to ventral sucker, generally overlapping right testis or/and right vitelline mass. Laurer’s canal not observed. Vitelline masses oval, paired, posterior to ventral sucker, situated on each side of body, symmetrical to oblique. Vitelline ducts joined medially posteriorly to ovary, forming thick common duct. Seminal receptacle oval, small. Uterus convoluted, with coils extending posteriorly between vitelline masses, passing dorsally to ventral sucker, barely visible as straight thin-walled tube, entering sinus-sac forming metraterm. Eggs elongate, oval. Excretory vesicle Y-shaped, bifurcating just behind vitelline masses; branches reuniting dorsally to pharynx.

Remarks: We found specimens in T. Odhner’s collections from *G. morhua*, *L. limanda* and *M. scorpius* that we re-examined. We attempted to detect any host-induced variations. Morphometric data are presented in Table 3. T. Odhner’s specimens from these three hosts share with our specimens of *D. varicus* the sausage-shaped body appearance and the organisation of the terminal genitalia. They differed by body length, forebody and hindbody length, ventral sucker size and size of the seminal vesicle. However, the number of measured specimens is low (one from *L. limanda*, 2 from *M. scorpius*, and 2 from *G*. *morhua*). In addition, from our examination, it is clear that T. Odhner flattened his specimens, which impairs morphometric comparison [12, 25]. However, we think it is likely that specimens collected by T. Odhner from *L. limanda*, *M. scorpius*, and *G. morhua* from Kristineberg are conspecific with our newly collected material from the Swedish coast of Skagerrak and off the coast of Norway, given the general morphological similarities and collection localities.

#### *Derogenes ruber* Lühe, 1900 (Figs. 9–12)

**Figure 9:**
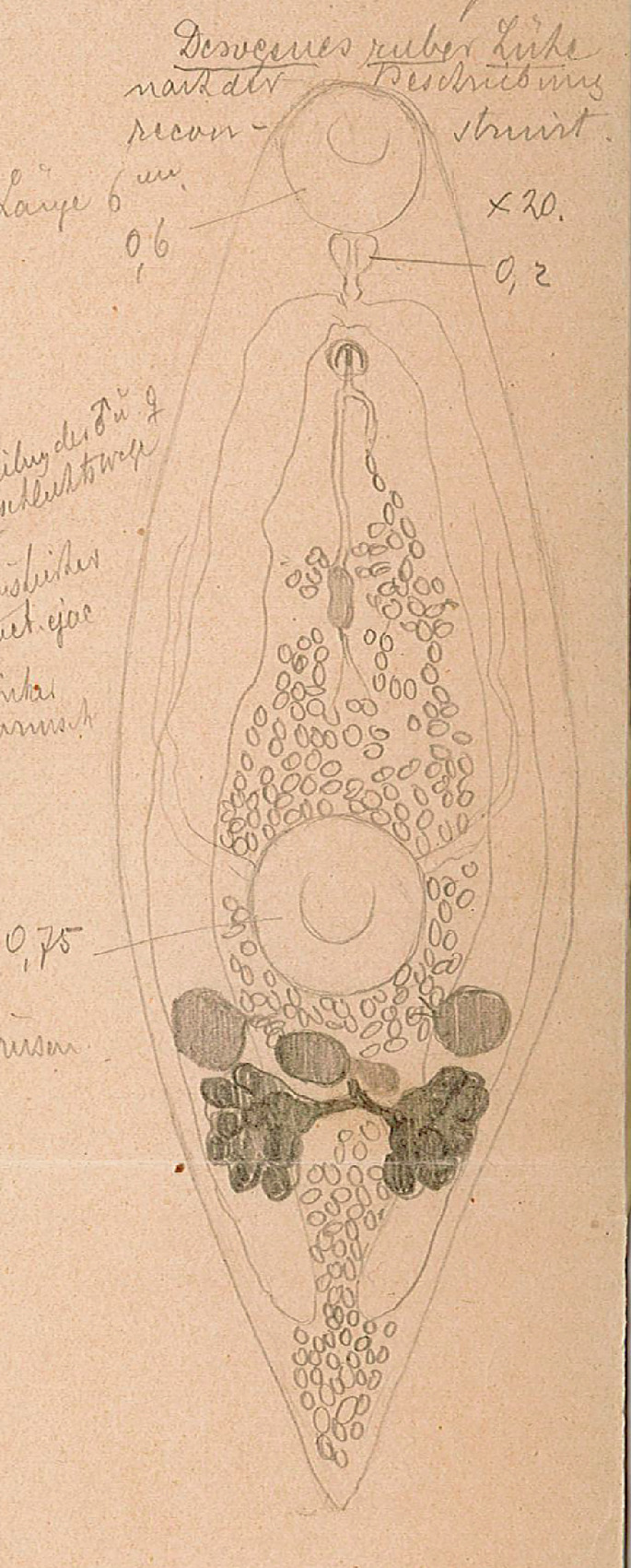
*Derogenes ruber* Lühe, 1900 ex *Chelidonichthys lastoviza*. Unpublished line drawing by A. Looss.

**Figure 10:**
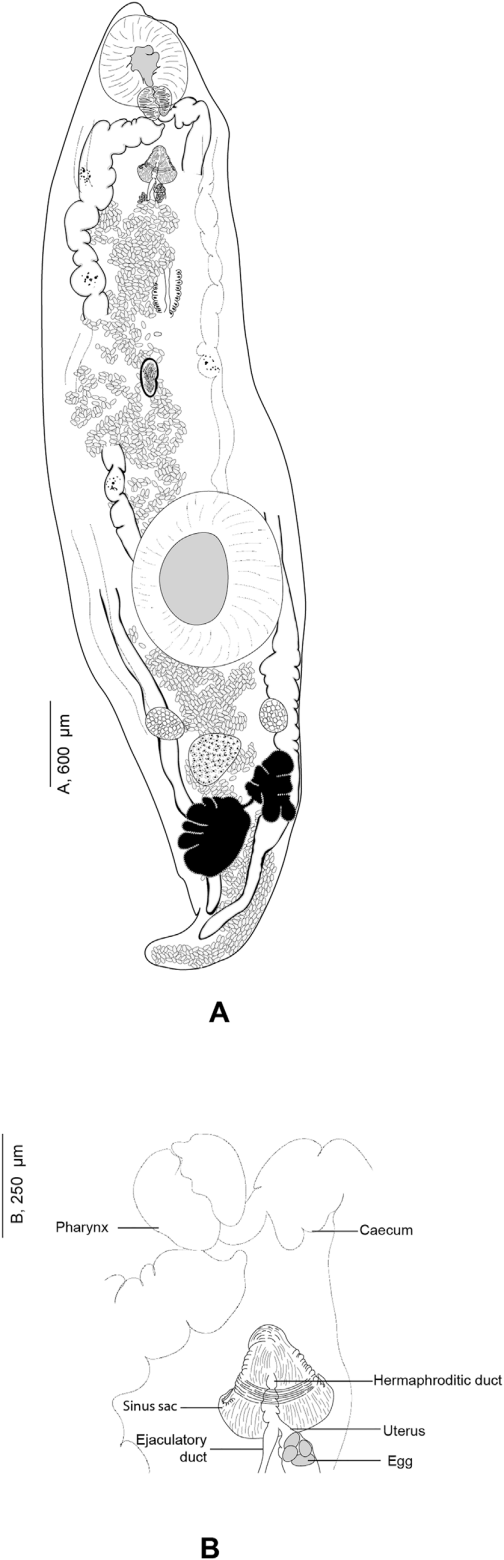
*Derogenes ruber* Lühe, 1900 ex *Chelidonichthys lastoviza* (SMNH-138743). A, Whole body. B, Terminal genitalia.

**Figure 11:**
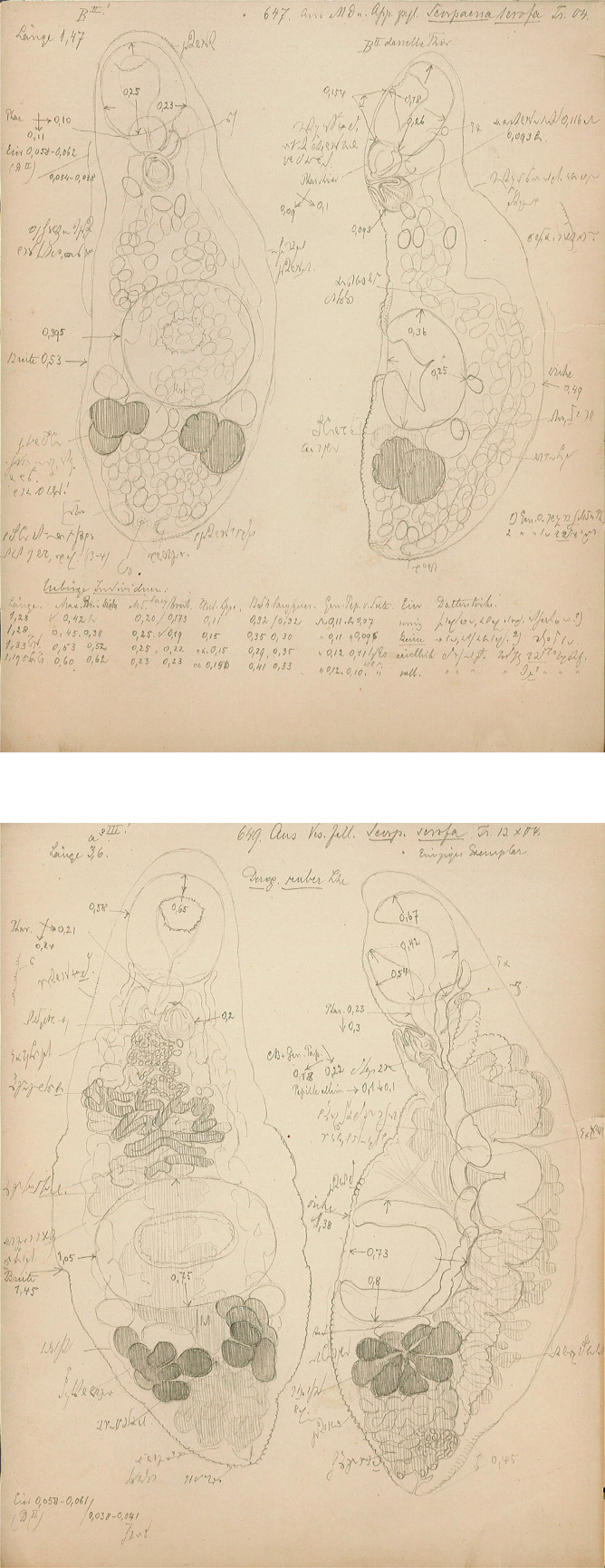
*Derogenes ruber* Lühe, 1900 ex *Scorpaena scrofa*. Unpublished line drawings by A. Looss.

**Figure 12:**
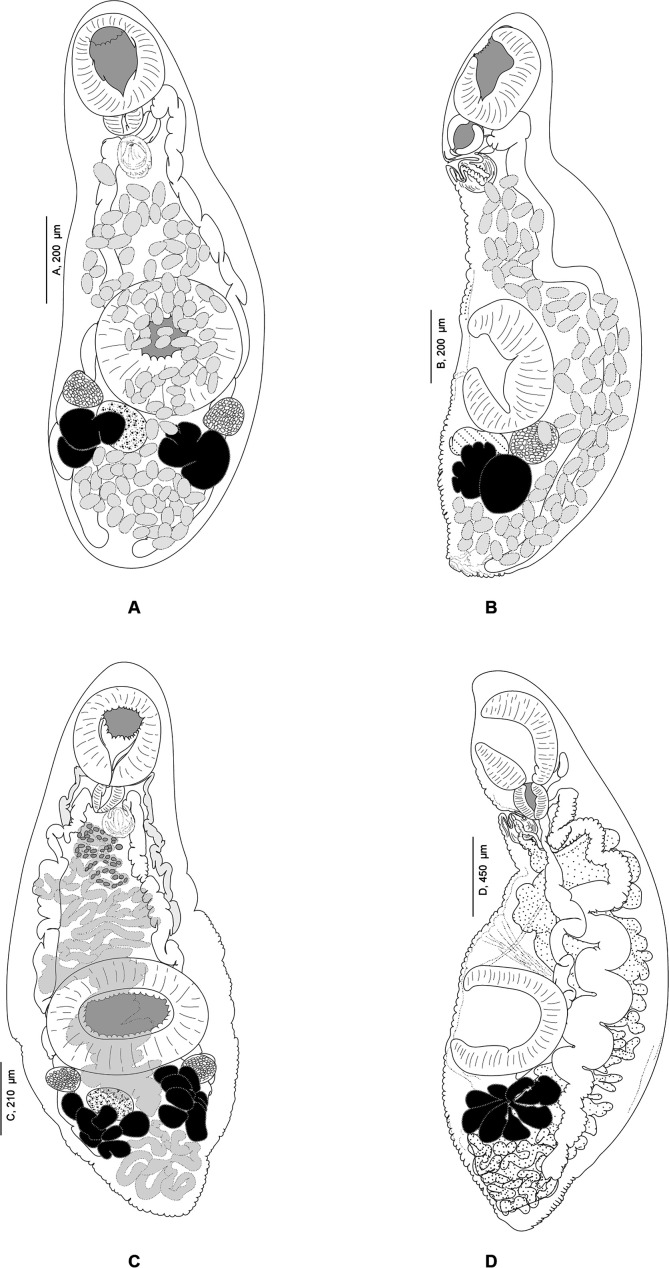
*Derogenes ruber* Lühe, 1900 ex *Scorpaena scrofa* based on A. Looss’s unpublished line drawings. A, Whole body, ventral view. B, Whole body, lateral view. C, Whole body, ventral view. D, Whole body, lateral view.

Type-host: *Chelidonichthys lastoviza* (Bonnaterre) (Perciformes: Triglidae) (as *Trigla lineata* Gmelin), the Streaked gurnard [47].

Type-locality: Rovinj, Croatia, Adriatic Sea, Central Mediterranean [47].

Site in type-host: Gall-bladder [47].

Other records:

Hosts: *Trigla lyra* (Perciformes: Triglidae), Piper gurnard [74].

Localities: Split, Croatia, Adriatic Sea, Central Mediterranean [74]. Azores, Canary and Cape Verde Isles [30] and Spain [10], Northeast Atlantic. Trieste, Italy, Western Mediterranean (present paper).

Site in host: Intestine (present paper).

Material examined: one slide (SMNH-138743) containing two adult specimens (originally numbered by A. Looss as 1818) of which one matches an unpublished line drawing of *D. ruber* (Fig. 9) found in A. Looss’s archive, collected from *C. lastoviza* on 12. 1900 in Trieste, Italy, Western Mediterranean (corresponding measurements of this specimen presented in Table 4) and four unpublished line drawings of *D. ruber* from *Scorpaena scrofa* Linnaeus (Fig. 11).

Measurements in Table 4. Body fusiform, voluminous. Posterior end blunt (Fig. 10A). Pre-oral lobe distinct. Oral sucker subterminal, subglobular. Prepharynx absent. Pharynx subglobular. Oesophagus short (Fig. 9). Intestinal bifurcation in anterior third of forebody. Caeca sinuous, difficult to trace in some body parts; blind termination of caeca clearly visible at posterior end. Ventral sucker voluminous, subglobular.

Testes small, symmetrical, in anterior hindbody. Seminal vesicle saccular, thin-walled. Pars prostatica tubular, lined by scattered prostatic glands. Sinus-organ muscular, conical, projecting into genital atrium (Fig. 10B). Sinus-sac muscular. Metraterm protruding along with ejaculatory duct into sinus-sac forming hermaphroditic duct. Genital pore posterior to pharynx (Figs. 11 and 12).

Ovary post-testicular, in anterior half of hindbody, situated at considerable distance behind ventral sucker, obscured by eggs. Uterine coils tightly packed with eggs, reaching posterior body end, distributed along entire hindbody; in forebody, uterine coils starting from anterior level of ventral sucker to short distance behind terminal genitalia. Metraterm muscular, joining male ejaculatory duct. Vitelline masses postovarian, paired, lobed. Eggs numerous, mostly collapsed in hindbody. Excretory vesicle not observed due to coiling of body posteriorly. Excretory arms visible in forebody; position of bifurcation of excretory arms not observed.

Remark: *Derogenes ruber* Lühe, 1900, the type-species of the genus, was first described from the tub gurnard *Ch. lastoviza* off the coast of Split, Croatia [47]. Being known only by the original description that lacked illustrations, we here provide a figure and additional information especially regarding the organisation of the terminal genitalia, based on two specimens found in Looss’s collection from the same host from off Trieste, Italy. Looss’s archive also included four drawings of *D. ruber* from *Scorpaena scrofa* Linnaeus (Fig. 11) that we present in Figure 12. The corresponding slides could not be found in Looss’s collection.

#### Derogenes minor Looss, 1901 (Figs. 13–14)

**Figure 13:**
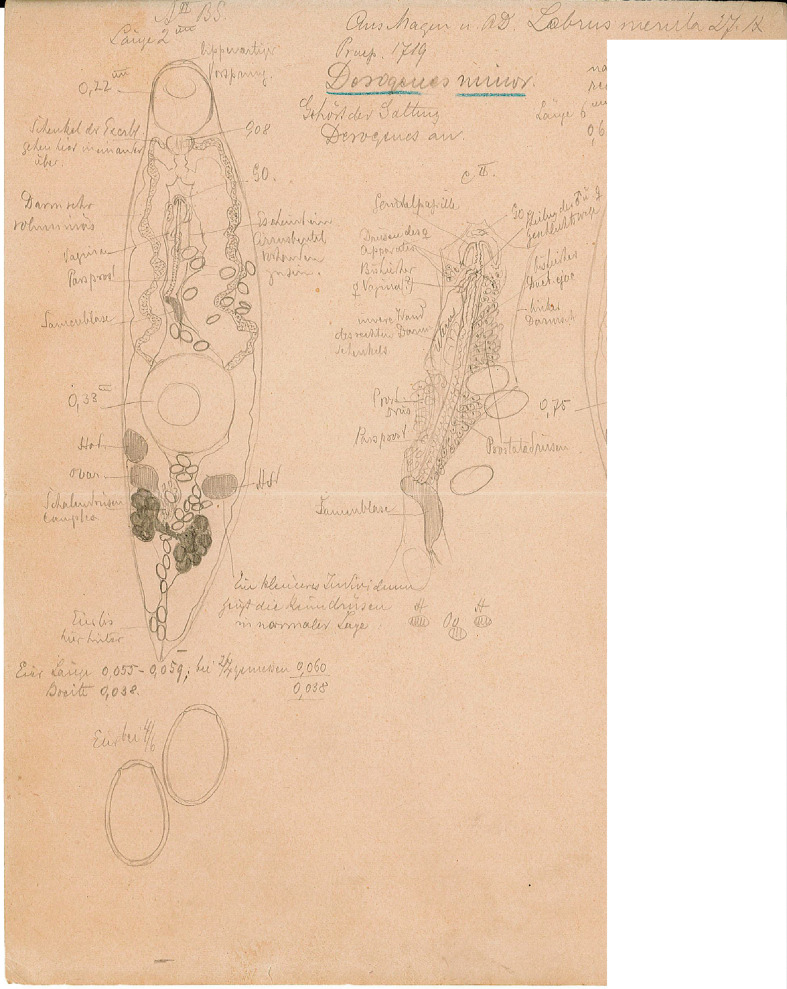
*Derogenes minor* Looss, 1901 ex *Labrus merula*. Original line drawing of *D. minor published* as Figure 5 in Looss (1901), and an unpublished line drawing of the terminal genitalia.

**Figure 14:**
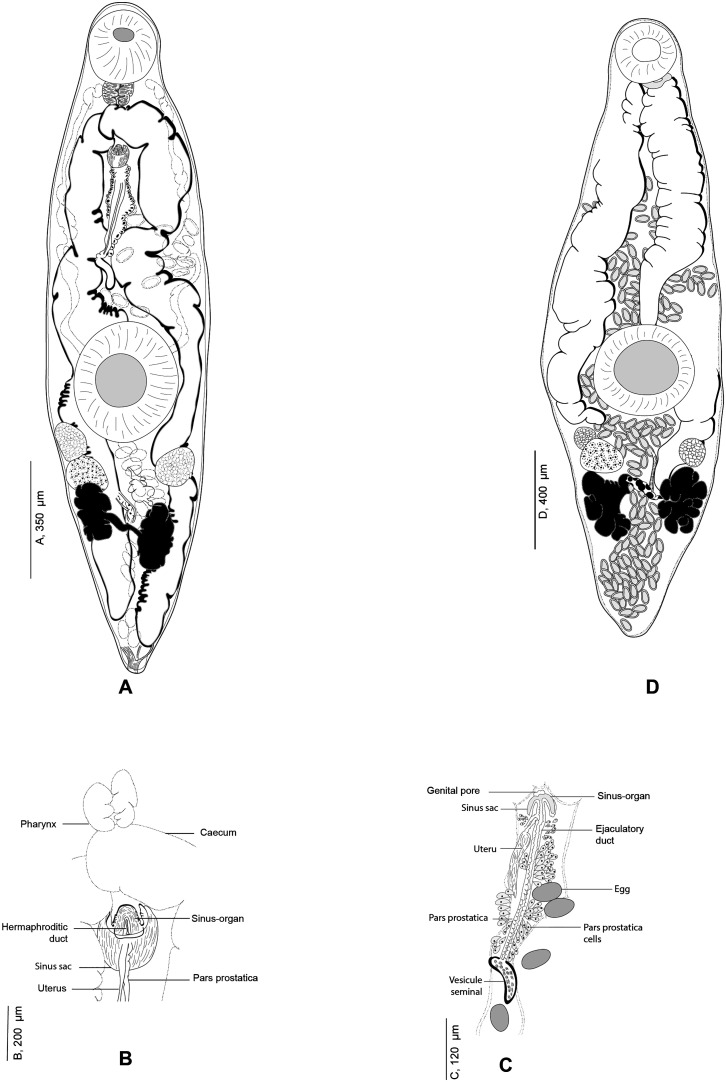
*Derogenes minor* Looss, 1901 ex *Labrus merula* and *Derogenes* cf. *minor* ex *Lophius budegassa*. A, *Derogenes minor* Looss, 1901 ex *L. merula*, Whole body, Lectotype (SMNH-138741). B, *Derogenes minor* Looss, 1901 ex *L. merula*, Terminal genitalia, Lectotype (SMNH-138741). C, *Derogenes minor* Looss, 1901 ex *L. merula* based on an unpublished line drawing by A. Looss. D, *Derogenes* cf. *minor* ex *Lophius budegassa*, Whole body (SMNH-138742).

Type-host: *Labrus merula* (Perciformes: Labridae), brown wrasse [45].

Type-locality: off Trieste, Italy, Western Mediterranean [45].

Site in type-host: Stomach [45].

Material examined: one slide containing two adult specimens designated as Lectotype (SMNH-138741). Additional material, specimens from *Labrus merula* preserved in ethanol, SMNH 892 currently in the wet collections of the SMNH, designated as Paralectotypes.

Material examined for comparison: Two specimens of *D.* cf. *minor* from the intestine of *Lophius piscatorius* Linnaeus, from Trieste, Italy, Central Mediterranean (SMNH-138742, SMNH-208362).

Archival documents: The archives include the original line drawing published as Figure 5 by Looss (1901), and an unpublished ink drawing of the eggs and of the terminal genitalia (see Fig. 13 here).

Measurements in Table 4. Body small, fusiform. Anterior end rounded, posterior end narrow and blunt (Fig. 14A). Pre-oral lobe short. Oral sucker subterminal, rounded. Prepharynx not observed. Pharynx muscular. Oesophagus short (Fig. 13). Intestinal bifurcation in anterior third of forebody, anterior to terminal genitalia. Intestinal caeca broad, convoluted, extending to posterior end, ending blindly close to posterior end of body. Ventral sucker muscular, rounded, voluminous.

Testes oval, near left and right margins of body posterior to ventral sucker. Seminal vesicle tubular, placed at short distance anteriorly to ventral sucker. Seminal vesicle followed by long pars prostatica, lined by small unicellular prostatic glands. Ejaculatory duct and metraterm visible passing into sinus-sac and forming hermaphroditic duct. Hermaphroditic duct thin, enclosed in sinus-organ, projecting into genital atrium (Figs. 14B and 14C). Genital pore opening medially, posteriorly to pharynx.

Ovary in hindbody, sinistral, close to body margin, slightly larger than testes. Seminal receptacle long, tubular, thin-walled, immediately anterior to vitelline masses. Laurer’s canal not observed. Vitelline masses postovarian, paired, compact, separated from one another. Excretory bladder Y-shaped: arms of excretory bladder reaching midlevel of pharynx. Eggs oval, thin shelled.

Remark: *Derogenes minor* is currently considered a valid species (WoRMS, 2022). The redescription here was based upon one slide (SMNH-138741) containing two adult specimens of which one matches the original description and illustration given by Looss (1901) (see his Fig. 5). Additionally, the sketching of *D. minor* found in A. Looss’s archive (see Fig. 13 here) matches the figure given in the original description as collected on 27.09.1900 off the coast of Trieste, Italy. This slide is thus designated as the Lectotype. Additional material, specimens from *Labrus merula* preserved in ethanol (SMNH 892) currently in the wet collections of the SMNH, are designated as Paralectotypes. Additional specimens include one specimen labelled as *D. minor* by A. Looss, from the Intestine of *Lophius piscatorius* Linnaeus, collected on 12.1900 off Trieste, Italy (SMNH-138742); one specimen labelled as *Derogenes* sp. by A. Looss, from the Intestine of *Lophius piscatorius* Linnaeus, collected on 10.1904 off Trieste, Italy (SMNH-208362) is designated here as *D*. cf. *minor* (Fig. 14D).

## Discussion

### Delimitation of *D. varicus sensu stricto*

*Derogenes varicus* has been considered the most common digenean species in fish [32]. It is widely reported in most of the oceans of the world as well as in freshwater systems. In the Northeast Atlantic, it has been recorded from more than 40 species of marine fish [49]. There are also some freshwater records, mainly in migratory diadromous fishes, such as salmonids [20]. The life cycle was described in detail by Køie [32]; naticid snails are the first intermediate hosts, releasing cystophorous cercariae that can infect calanoid copepod, among which only *Calanus* spp. may act as second intermediate hosts. In definitive hosts, the adults are usually found in the oesophagus or stomach.

Several authors have suggested that *D. varicus* comprises two or more species and Køie [34] suggested that *D. varicus* from the northeast Atlantic should be referred to as *D. varicus* *sensu lato* or as *Derogenes* sp. A recent comprehensive study by Krupenko *et al.* [35] provided evidence that *D. varicus* can be genetically split. Four genetic groups were recognised, independent of locality or host to a certain extent: *D. varicus* DV1 occurring in *G. morhua*, *M. scorpius*, *L. limanda*, *A. lupus*, *E. nawaga* and the Pacific herring *C. pallasii*, *D.* cf. *varicus* DV2 from *H. platessoides*, *D.* cf. *varicus* DV3 from *E. fedorovi* Mandrytsa, 1991, and *D.* cf. *varicus* DV4 from *H. platessoides* (based on 18S sequences). Herein, all the newly generated 28S sequences of *D. varicus* from fishes collected off the coast of Sweden and Norway, including that from a specimen from the type-host *S. salar* were identical, and clustered within the *D. varicus* DV1 clade. Although the type-locality of *D. varicus* is Danish marine waters, our collections off the coast of Sweden essentially encompass this area. Additionally, Müller [58] did not specify a locality from which he collected the salmon infected by “*Fasciola varica*”. He mostly worked in Øresund in Denmark and in Drøbak in the Oslofjord, Norway. It seems most likely that the studied salmon originated from the Øresund salmon fishery. Thus, we consider the clade identified as *D. varicus* DV1 by Krupenko *et al.* [35] to represent the true *D. varicus*, herein designating it as “*D. varicus sensu stricto*”.

Our *D. varicus* *sensu stricto* specimens from *M. merlangus* were morphologically similar to that of *D. varicus* *sensu lato* from *L. limanda*, *G. morhua* and *M. scorpius* from T. Odhner’s collection, which supports the presence of a single species in the *D. varicus* DV1 lineage. More importantly, the intraspecific genetic variations between members of the *D. varicus* DV1 (i.e., from different host species) were of an order of magnitude lower than the interspecific divergence between members of *D. varicus* DV1 and the well established species *D. lacustris*: 0% *vs*. 8–9% (68 substitutions) in 28S, 0–1% *vs*. 19% in *cox*1. The divergence of *cox*1 reported herein for *D. varicus sensu stricto* agrees with intraspecific variations, which are typically below 6.0% in Digenea [82] and the genotypes were independent of host species or localities. Among Derogenidae, previously reported interspecific divergences in *cox*1 ranged between 10.5–15.1% [83] and 16.9–20.4% [83], while in other lineages divergence reached 8.9–26.5% [6]; 9.6–12.8% [4]; 9.9–15.1 [44]; and 20% [26]. Hence, divergences reported within the DV1 clade appear consistent with intraspecific divergences. Consequently, we consider the lineages DV2, DV3 and DV4 to represent different species. In light of the available data, *D. varicus sensu stricto* (DV1) is euryxenous. Hence, this work is a step towards untangling this species complex and delimiting the cryptic complex hidden under the single name “*D. varicus*”.

We compared our *D. varicus* *sensu stricto* from *S. salar*, *M. merlangus* and from *G. morhua* from Sweden and Norway, with specimens from T. Odhner’s collection (Table 3). However, T. Odhner’s specimens were excessively flattened and thus to consider any differences as intraspecific variations is unreliable. Additionally, they had similar morphology and anatomy (Figs. 7 and 8). *Derogenes varicus* from *M. merlangus* and from *G. morhua* from Scandinavian waters are genetically identical to *D. varicus* *sensu stricto* from various hosts (see [35]). They were also morphologically similar as measurements overlapped (Table 3). Additionally, they showed no divergence in 28S or ITS2 sequences and differed only by 0–1% in the *cox*1 gene region.

Problems arise with species identification within the *D. varicus* complex because several species of *Derogenes*, including the type-species, were described only superficially [47], and data on taxonomically important characters of the “true” *D. varicus* were incomplete. The original description of *D. varicus* does not allow consideration of several important morphological characteristics such as the extent of the intestinal caeca, position of the genital pore, relative position of the ovary and testes, and several other features, some of which are now used to distinguish *Derogenes* species. Consequently, the redescriptions and delimitation of *D. varicus sensu stricto* given herein are crucial for untangling the species complex in the future to describe and discriminate the cryptic species detected in *D. varicus sensu lato*.

### Other species within *Derogenes*

The type-species of the genus, *D. ruber* Lühe, 1900, was first described from the gall bladder of the streaked gurnard *Ch. lastoviza* (as the syn. *Trigla lineata*) from off the coast of Roving, Croatia, Adriatic Sea [47]. It was redescribed from the piper gurnard *Trigla lyra* from off the coast of Split, also Croatia [74]. We noted some minor morphometrical differences between the two Adriatic records: *Derogenes ruber* from *T. lyra* differed from *D. ruber* from the type-host *Ch. lastoviza* by having a smaller body (4200–4500 × 1300–1800 *vs*. 5000–6000), smaller ventral sucker (950–1290 *vs*. 750), smaller pharynx (168 *vs*. 200) and ovary (252 *vs*. 450). However, the number of measured specimens is low (2 specimen were measured in both studies) [47, 74]. Similarly, specimens of *D. ruber* from *Ch. Lastoviza* from Italy examined herein differed slightly from those from the same host, from the type locality Croatia, by having a larger body (7869 × 1847 *vs*. 5000–6000 × 2000), larger oral sucker (655 × 708 *vs*. 505) and larger pharynx (200 *vs*. 168). We also note that despite sharing the same host, *Ch. lastoviza*, *D. ruber* of Lühe [47] were collected from the gall-bladder while the two specimens examined here were collected from the intestine. We do not know if this is a post-mortem migration of *D. ruber* as the gall-bladder and the intestine (and also pyloric caeca) were regarded as unusual sites for derogenids, which are normally stomach parasites [2]. Additionally, *D. ruber* from *T. lyra* reported by Sey [74] and from the present specimens by having strikingly smaller eggs (23 × 23). However, Bartoli and Gibson [2] pointed that this egg-size value is presumably an error.

A species very similar to *D. ruber* is *D. latus* Janiszewska, 1953, first described based on a single specimen from the intestine of the red mullet *Mullus barbatus* from the same Adriatic locality as that of *D. ruber*, off Split, Croatia [27].

*Derogenes latus* was redescribed from the intestine of *M. barbatus* and *Trisopterus capelanus* (Lacépède, 1800) in the North Adriatic Sea [64] and from the gall-bladder of *M. surmuletus* off Corsica (France), Western Mediterranean [2]. The redescription provided by Bartoli and Gibson [2] based on accessible voucher material and serial sections provided several morphological and anatomical details along with morphometrical data. *Derogenes latus* was frequently reported from its type host from the Western Mediterranean, off Spain [16] and off France [38], and from a closely related host, *M. surmuletus* from the Western Mediterranean (off France and Algeria [7, 23, 38, 81]).

It was also reported on hosts other than Mullidae, mainly from *S. scrofa* (Scorpaenidae) from the Western Mediterranean, off Spain [46] and off France [81]; from *L. mormyrus* (Sparidae) off Montenegro, Adriatic Sea [68] and off Algeria, Western Mediterranean [5]. It was furthermore recounted from *Sardinella aurita* Valenciennes. (Dorosomatidae) off Algeria, Western Mediterranean [69] and from *Phycis phycis* (Linnaeus) (Phycidae) from the Western Mediterranean (off France) [81]. *Derogenes latus* was also reported in a Triglidae, *Chelidonichthys cuculus* (Linnaeus, 1758) [61]. However, triglids are commonly considered hosts of *D. ruber* [2].

Curiously, we found in Looss’s archive unpublished lined drawings of derogenids identified as *D. ruber*, from *S. scrofa*, mentioned also as a host for *D. latus* [46] and off France [81]. We could not find the slides used to make these illustrations. Despite having different hosts, *C. lastoviza* for *D. ruber* [47] and *M. barbatus* for *D. latus* [27], the distinction between *D. ruber* and *D. latus* has been questioned [2]. *Derogenes ruber* and *D. latus* share a stout body, post-testicular vitellarium composed of two multilobed masses and a uterus occupying almost the entire body [2, 64, 68]. We have confirmed these features in specimens from A. Looss’s collections, and illustrations of *Derogenes ruber* and *D. latus* clearly share the previously mentioned anatomical features.

Although recent studies have used molecular sequence data to test the putative broad host specificity of some species of *Derogenes* and both *D. lacustris* [82] and *D. varicus sensu stricto* have been shown to occur across a wide variety of hosts (see Figs. 2–4), as mentioned above, even if *D. ruber* is known only from its type host, a Triglidae (Perciformes), the closely related species *D. latus* was reported from 6 species, belonging to 5 families (Mullidae, Scorpaenidae, Sparidae, Dorosomatidae, and Phycidae) across 5 orders (Mulliformes, Scorpaeniformes, Spariformes, Clupeiformes, and Gadiformes) and consequently, synonymising the two species without supporting molecular data will make *D. ruber* a euryxenous species occurring in hosts across 7 families and across 6 orders. Such a nomenclature act without supporting molecular data might lead to making *D. latus* the Mediterranean version of “*D. varicus*”. Although Bartoli and Gibson [2] highlighted marked morphological similarity between *D. latus* and *D. ruber* and convincedly argued about possible synonymy between the two species, they refrained from synonymising the two species formally until further studies of material from the type-hosts and localities are available. Molecular data of *D. ruber* have recently been made available [18], but we refrain from synonymising the two species until molecular data are available for *D. latus*.

Hence, in light of available data, we can only affirm that *D. ruber* and *D. latus* Janiszewska, 1953 are very similar, apparently differing only in the degree of lobation in the vitelline masses. They do differ in principal host and site and taking into consideration the potential synonymy between the two species suggested (Bartoli and Gibson [2]), molecular studies on new material are needed to clarify this issue. Additionally, we could unfortunately not examine any *D. latus* and thus the validity of this species cannot presently be further considered.

*Derogenes minor*, currently a valid species [21], was first described from the brown wrasse *Labrus merula* (Labridae) from off the coast of Trieste, Italy [45]. Although some ambiguity surrounded the collections of A. Looss after leaving Egypt at the beginning of World War I, part of his collections was sold to Dr. Theodor Odhner, a former Professor at the Department of Invertebrate Zoology at the Museum of Natural History (Naturhistoriska riksmuseet) in Stockholm by his widow [36, 62]. The archive of the SMNH includes a list of material of A. Looss, including hosts, locality, date, publication-ready ink drawings and unpublished line drawings. The specimens of *D. minor* we found in A. Looss’s collection agree with the original description and one of the two specimens examined matches the line drawing found in the archive of A. Looss and the drawing published as Figure 5 by Looss (1901). Hence, this is clearly the type-material of *D. minor.* We here supplement the original description [45] by providing additional morphometrical data and with a description of the organisation of the terminal genitalia based on re-examination of type specimen.

In A. Looss’s collection, we also found two putative specimens of *D. minor* from the intestine of the angler, *Lophius piscatorius,* caught off the coast of Trieste, Italy. One of them lacking the posterior part is labeled as *D. minor* and the second one is entire and labelled by A. Looss as *Derogenes* sp. (Fig. 14D). Measurements of *D. minor* from *L. piscatorius* and those we examined from *L. merula* overlapped. The specimen labelled as *Derogenes* sp. from *L. piscatorius* differed slightly from *D. minor* from the type-host *L. merula* by having a larger body and larger vitelline masses. They shared, however, the lobed vitelline masses, specifically the mulberry-like shape, a characteristic of *D. minor* [45]. This situation is puzzling as *L. piscatorius* was reported as a host of *D. latus* Janiszewska, 1953 [2, 3]. However, *D. latus* and *D. minor* are supposed to be distinguished partially by the shape of the vitelline masses, being mulberry-like in *D. minor* [45] vs. deeply lobed or lobed in *D. latus* [27]. The vitelline masses of the specimen we examined from *L. piscatorius* (Fig. 14D) appear intermediate between the previously mentioned shapes; it appears mulberry-like with deep lobes but resembles *D. minor* in its narrow hindbody. Hence, as we examined only one specimen, on which unfortunately the terminal genitalia could not be observed, we identify the derogenids examined from *L. piscatorius* as *D.* cf. *minor* pending further study.

**Figure 15:**
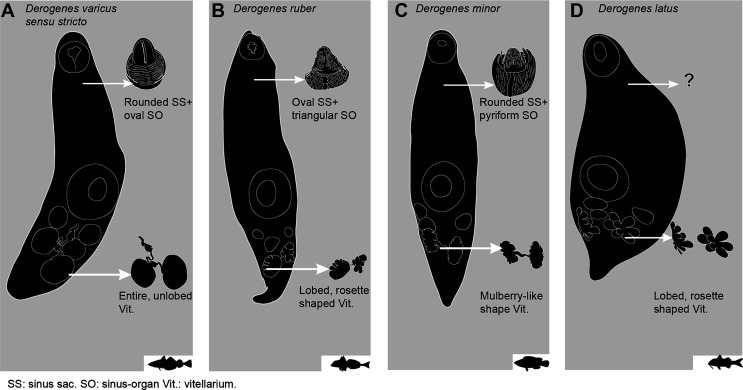
Diagram showing the similarities and differences between some *Derogenes* species. A, *Derogenes varicus* (Müller, 1784) *sensu stricto* (SMNH-9563). B, *Derogenes ruber* Lühe, 1900 (SMNH-138743)*.* C, *Derogenes minor* Looss, 1901 (SMNH-138741). D, *Derogenes latus* Janiszewska, 1953.

A diagram comparing four species of *Derogenes* (Fig. 15) suggests that the organisation and shape of vitelline masses is the most straightforward character to distinguish *Derogenes* species. In *D. varicus sensu stricto*, the vitelline masses are unlobed (Fig. 15A). In *D. ruber*, the vitelline masses are lobed and rosette shaped (Fig. 15B). In *D. minor*, the vitelline masses are mulberry-like shaped (Fig. 15C). In *D. latus*, the vitelline masses are deeply lobed, and rosette shaped (Fig. 15D). We also attempted to test the utility of the terminal genitalia, and in *D. varicus sensu stricto*, the sinus-sac is rounded (Fig. 15A) and the genital atrium is almost half the length of the sinus-sac, and the basal musculature of the sinus-sac does not reach the level of the genital atrium. In *D. ruber*, the sinus-sac is ovoid and elongated transversally (Fig. 15B), and the genital atrium is two-thirds of the length of the sinus-sac, and the musculature is weakly developed and extends only to the base of the genital atrium. In *D. minor*, the sinus-sac is rounded (Fig. 15C), and the genital atrium is small, only about one third the length of the sinus-sac and the musculature covers the genital atrium. However, the three species share a conical sinus-organ and most importantly, the previously mentioned differences are high, likely affected by the wall musculature contraction, and should be taken with caution when distinguishing species. Unfortunately, we could not examine specimens of *D. latus* to comment further on the form of the terminal genitalia. Hence, it would be valuable to investigate these features, especially the form and size of the sinus-organ, to test their utility in species delimitation. Although the striking morphological similarities between *D. latus*, *D. ruber* and *D. minor* suggests possible synonymy between the three [2], especially with respect to the large host spectrum for *D. varicus* supported here with molecular data, we refrain from making any taxonomic proposals regarding the Mediterranean specimens as molecular data are lacking and we consider them valid species pending further studies. The following species have previously been synonymised with *D. varicus*: *Anisocoelium hippoglossi* MacCallum, 1921, *D. fuhrmanni* Mola, 1912 and *D. parvus* Szidat, 1950 [85]. *Anisocoelium hippoglossi* MacCallum, 1921, described from the Atlantic halibut *Hippoglossus hippoglossus* (Linnaeus, 1758) collected off the coast of Massachusetts, USA exhibits unique characters relative to *D. varicus sensu stricto*, with the ovary a considerable distance in front of the ventral sucker [48]. If these features were correctly characterised, then *A. hippoglossi* MacCallum, 1921 is not a synonym of *D. varicus sensu lato* nor even a species of *Derogenes*. Since the species appears to never have been recollected, it could be that the seminal vesicle of a derogenid was taken for the ovary, or that the specimen was teratological. *Derogenes fuhrmanni* Mola, 1912, was first described from the European bullhead *Cottus gobio* Linnaeus from River Aniene, in Lazio, Italy and additional specimens brought from Rome as Mola stated “*In etwa 30 Exemplaren von Cottus gobio, wovon einige aus dem Flusse Aniene stammten und andere von mir in Rom*” [56]. It was subsequently synonymised with *D. varicus* [67]. In addition to the hosts being different (*Cottus gobio*, Cottidae for *D. fuhrmanni* *vs*. *Salmo salar*, Salmonidae for *D. varicus*) and especially the localities being widely separated (River Aniene, Italy for *D. fuhrmanni* *vs*. Denmark for *D. varicus*), *Derogenes fuhrmanni* also differs from *D. varicus* by having a larger body (4000 × 1000 *vs*. 2276 × 528), larger ventral sucker (600 *vs*. 353 × 374), and strikingly larger eggs (72 × 27 *vs*. 40–53 × 28–35). We consider thus *D. fuhrmanni* as a valid species. We also examined the original description of *D. parvus* first described from the Patagonian blennie *Eleginops maclovinus* (Cuvier, 1830) off the coast of Tierra del Fuego, southeastern Atlantic [79]. It can be distinguished from *D. varicus* *sensu stricto* by the testes, the ovary, and the globular parts of the vitelline masses being all the same size, and by having a much larger ventral sucker (697 *vs*. 300 × 299). The most distinctive feature distinguishing *D. parvus* and *D. varicus sensu stricto* is the vitelline masses being divided into numerous small lobes in *D. parvus* [79]. The possession of lobed vitelline masses might suggest possible synonymy with *D. minor* or with *D. latus* and *D. ruber*. However, Szidat (1950) stated that the vitelline masses are far smaller in *D. parvus* than those described by Looss in *D. minor* [79]. We therefore consider *D. parvus* a distinct and valid species.

## Conflict of interest

Authors have no potential conflict of interest.
